# Nutrient-induced germination of *Bacillus subtilis* spores exhibiting shared metabolic profiles in both TSB and AGFK media

**DOI:** 10.1007/s11306-026-02442-4

**Published:** 2026-05-16

**Authors:** Nana Asiamah Boateng, V. M. Balasubramaniam, Melvin A. Pascall, Matthias S. Klein

**Affiliations:** 1 Department of Food Science and Technology, The Ohio State University, 2015 Fyffe Road, Columbus, OH 43201 USA; 2 Department of Animal Science, McGill University, 21111 Lakeshore Road, Sainte-Anne-de-Bellevue, QC H9X3V9 Canada

**Keywords:** *Bacillus subtilis*, Germination, Sporulation, Tryptic Soy broth, Metabolomics, AGFK

## Abstract

**Introduction:**

*Bacillus subtilis* is a spore-forming bacterium commonly implicated in food spoilage and foodborne illnesses due to its resistance to harsh conditions. Upon exposure to favorable environments, the spores germinate and resume metabolic activity and thus pose a risk to food safety. Detecting early germination stages is thus crucial for preventing contamination and subsequent illness outbreaks.

**Objectives:**

This study evaluated the potential of nuclear magnetic resonance (NMR)-based metabolomics to identify significant metabolites released during *Bacillus subtilis* OSU 494 spore germination using two nutrient germinants. The goal was to uncover biomarkers that could support improved detection strategies in food safety applications.

**Methods:**

The spores were induced to germinate using either tryptic soy broth (TSB) or L-asparagine, D-glucose, D-fructose, and potassium chloride (AGFK). The samples were collected hourly over 4 h and analyzed using an 850 MHz NMR spectrometer with a triple-resonance cryoprobe. 1D-¹H NOESY and 2D ¹H–¹³C HSQC spectra were obtained. Spectral binning and linear modeling were then applied to identify significant metabolic features.

**Results:**

The AGFK-induced germination yielded dipicolinic acid (DPA), L-alanine, acetic acid, L-phenylalanine, and formic, succinic, and fumaric acids. The TSB-induced germination produced DPA, L-alanine, L-phenylalanine, acetic and fumaric acids.

**Conclusion:**

Several metabolites were consistently released during germination in both nutrient conditions. These metabolites, particularly DPA and L-alanine, served as reliable biomarkers for the *Bacillus subtilis* spore germination. They then provided valuable insights for developing rapid detection tools to enhance food safety monitoring and contamination control.

**Supplementary Information:**

The online version contains supplementary material available at 10.1007/s11306-026-02442-4.

##  Introduction

Spore-forming bacteria, primarily members of the *Bacillus* and *Clostridium* genera, are rod-shaped organisms which are classified within the phylum *Firmicutes* (Galperin, 2016; Abecasis et al., 2013). These organisms are capable of forming endospores, a survival strategy that allows them to endure harsh environments (Setlow, 2010; Cortezzo & Setlow, 2005). Spore formation is triggered by unfavorable conditions like nutrient depletion, extreme temperatures (such as thermal processing), or high-pressure processing (Setlow, 2006; Zhang et al., 2018). Spore forming bacteria gain their dormancy (metabolic inactivity) and resistance to harsh conditions through a process called sporulation in which they transform from vegetative cells into spores within a few minutes (Hutchison et al., 2016). This allows them to survive for as long as the conditions persist, which may be several years (Ulrich et al., 2018). When the environment becomes favorable again, the spores germinate and transform back into metabolically active vegetative cells, ready for growth and reproduction.

Spore-to-cell transformation is a multi-step progression that includes activation, germination, and outgrowth. This series of changes is often described as “germination.” The ability of spore-forming bacteria to form spores and germinate under unpredictable conditions presents a significant challenge to the food industry (Carlin, 2011). In certain situations, bacterial spores can survive heating and dehydration. Within many food products, favorable conditions can trigger the spores’ germination, leading to rapid bacterial multiplication and food spoilage (André et al., 2017; Sharma et al., 2021). Additionally, some germinated bacteria can produce toxins that are responsible for foodborne illnesses (Bennett et al., 2013).

Bacterial spore germination has been extensively studied in the past and stages of germination have been defined using qualitative and phenotypic data, such as gene expression, protein analysis and microscopic imaging (Paredes-Sabja et al., 2011; Setlow, 2014). However, there is a gap in knowledge about the metabolic activities of these organisms during the germination process.

Metabolomics is a novel approach used to analyze changes in cell metabolism by measuring complex mixtures of metabolites combined with computational data analytical methods (Klassen et al., 2017). Applications of metabolomics extend far beyond food science laboratories. Examples include evaluations of the efficacy and safety of drugs, identification of disease biomarkers, and investigating the impact of dietary components on metabolic health (Hunter, 2009). Metabolomics allows us to identify mechanistic connections between diet and disease progression, thereby facilitating the development of personalized medicine and targeted nutritional therapies. Thus, metabolomics is rapidly transforming our understanding of biological systems across various disciplines (Wishart, 2008; Wilkins & Trushina, 2018; Chin & Slupsky, 2013). However, little is reported in the literature about the use of metabolomics as a tool in understanding the bacterial spore germination process. Previously published reports have shown that metabolomics (or related techniques) can be used to detect and/or identify dormant bacterial spores (Goodacre et al., 2000; Correa & Goodacre, 2011). According to Yan et al. (2015), 53 compounds were identified from fermentation caused by *Bacillus licheniformis* using ^1^H Nuclear Magnetic Resonance (NMR) based metabolomics. Also, NMR metabolomics is highly reproducible and requires minimal sample preparation (Markley et al., 2017). Proton NMR-based metabolomics has been employed to identify food microbes based on secreted metabolites (Wang et al., 2021). These studies demonstrate the potential of NMR-based metabolomics as a technique for analyzing biochemical profiles of selected bacteria and spores.

A good understanding of the molecular processes involved in spore germination would be an advantage in developing strategies designed to mitigate foodborne diseases and spoilage driven by spore forming bacteria. Therefore, the aim of this study was to identify metabolites released by germinating *Bacillus subtilis* OSU 494 using Tryptic soy broth (TSB) and L-asparagine, D-glucose, D-fructose, and potassium chloride (AGFK) as nutrient germinants. *Bacillus subtilis* was selected because it has been extensively studied, and it serves as a surrogate for pathogenic spore-formers such as *Bacillus cereus* and *Clostridium botulinum* (Setlow, 2014).

## Materials and methods

*Bacillus subtilis* strain OSU 494 was obtained from Dr. Ahmed Yousef’s laboratory in the Department of Food Science and Technology at The Ohio State University, Columbus, Ohio, United States.

### Media and buffer preparation

All media solutions were prepared as specified by the manufacturer. For tryptic soy agar (TSA), 30 g of TSB powder (BD Bacto™, Franklin Lakes, NJ) and 15 g of agar powder (BD Bacto™, Franklin Lakes, NJ) were measured and added to a beaker containing 1 L of deionized water. For TSB, a 30 g aliquot of TSB powder was added to 1 L of deionized water. Both media were heated to 60 ℃ and stirred at 800 rpm for 10 min. The mixtures were then autoclaved at 121 ℃ and a pressure of 103.4 kPa for a duration of 20 min. Potassium phosphate buffer was prepared at pH 7.0 by weighing 93.434 mg of potassium phosphate dibasic and 63.09 mg of potassium monobasic salt into a beaker containing 20 mL of sterile deionized water. The mixture was stirred at 25 ℃ until fully dissolved.

### Germinant solution preparation

The AGFK germinant solution was prepared using a method described by Porębska et al. (2016) with slight modifications. Individual stock solutions of L-asparagine, D-glucose, D-fructose, and potassium chloride were prepared by separately dissolving 0.16 g, 0.22 g, 0.22 g, and 0.09 g of each compound in 40 mL of deionized water, respectively, to obtain a final concentration of 30 mM. Each solution was sterilized by filtering using a membrane having a pore size of 0.22 μm. From these stock solutions, 2 mL of each L-asparagine, D-glucose, D-fructose, and potassium chloride were mixed with 2 mL of potassium phosphate buffer (pH 7) to give 10 mL of AGFK germinant solution. These were then stored in the refrigerator at 4 ℃ until ready for use.

### Sporulation media preparation

A method described by Wuytack et al. (2000), with slight modifications, was used to prepare the sporulation medium. A total of 11.5 g nutrient agar powder, 0.25 g potassium dihydrogen phosphate (KH_2_PO_4_), and 0.06 g magnesium sulphate (MgSO_4_) were mixed with 500 mL of deionized water and stirred at 60 ℃ until fully dissolved. The medium was autoclaved at 121 ℃ and 103.4 kPa for 20 min. It was then cooled to room temperature (25 ℃) before use.

### Spore crop preparation

The spore crops were prepared using a method reported by Wuytack et al. (2000) with slight modifications. A frozen stock culture of *Bacillus subtilis* OSU 494 stored at −80 ℃ was revived by thawing. An inoculum from this stock was streaked onto a TSA plate and incubated aerobically at 37 ℃ for 24 h. Cells grown on this TSA plate were then inoculated onto the sporulation medium and incubated aerobically at 37 ℃ for 7 days. A phase contrast microscopy was then used to confirm that over 90% of the cells had converted into spores. These spores were then transferred into sterile 50 mL centrifuge tubes (VWR International Radnor, PA) by flooding the plates with cold sterile deionized water and scraping the surface with a sterile inoculating loop. The spore suspension was then washed with sterile deionized water followed by centrifugation at 4 ℃ and 8000 rpm for 15 min. The supernatant was discarded and washing of the pellets was repeated three times. This was then sonicated at 25 ℃ for 10 min and the spore pellets resuspended in sterile deionized water to obtain approximately 10^8^ spores/mL (Ratphitagsanti et al., 2010). It was then stored at 4 ℃ until further analysis.

### Germination experiment

Before the germination experiment, the spore suspension was heat activated at 80 ℃ for 15 min in a water bath (Luu et al., 2015). It was then immediately cooled on ice for at least 10 min before being ready for the germination experiment. The germination status was assessed by observing changes in the spores from phase-bright to a phase-dark appearance using the phase contrast microscope (Supplemental Figure S16).

#### TSB induced germination

A 10 mL aliquot of the heat shocked spore suspension was added to 10 mL of the TSB germinant. The mixture was then incubated at 37 ℃ for 4 h. At 0, 1, 2, 3, and 4-hour time points, 5 mL aliquots of the mixture were sampled. The collected samples were filtered using Amicon ultra centrifugal filters (Millipore Sigma, Burlington, MA) with a 10 kDa molecular weight cutoff, to remove macromolecules, spores, and bacteria.

#### AGFK induced germination

Germination was induced by mixing 10 mL of heat activated spore suspension with 10 mL of the AGFK germinant solution (Pandey et al., 2013), followed by incubation at 37 ℃ for 4 h. Aliquots of 5 mL of the mixture were collected at 0, 1, 2, 3 and 4-hour time points. The samples were filtered using filter units with a pore size of 0.22 μm to remove spores and bacteria.

### Sample preparation and measurements

Six biological replicates of growth media were collected for each time point. Deuterium oxide (D_2_O), trimethylsilyl-2,2,3,3-tetradeuteropropionate (TSP) (as an internal standard) and boric acid as an antimicrobial were added in a ratio of 12 parts sample to 1-part D_2_O mixture, and the pH was adjusted to 7.4. Boric acid was used to prevent growth of potentially remining vegetative cells. Aliquots of 650 µL from each sample were transferred to 5 mm NMR tubes (Norell, Morganton, NC) and immediately transferred to the NMR spectrometer after the 4-hour time point collection. The samples were kept at room temperature (25 °C) before analysis on the 850 MHz Avance III HD Ascend spectrometer equipped with a triple-resonance inverse cryoprobe and z-gradients (Bruker BioSpin, Billerica, MA). All samples were measured at 298 K and automatically locked, tuned, matched, and shimmed. For all samples, 1D ^1^H NOESY spectra were collected, and a 2D ^1^-^13^ C HSQC spectrum was collected for one selected sample. The 1D spectra were used to identify signals that increased significantly during germination, while the 2D spectrum was used to help in identification of the molecules that gave rise to the signals observed in the 1D spectra.

###  NMR data processing

All spectra were manually phased, baseline corrected, and the chemical shifts referenced to the TSP, using the Topspin 4.14 software of the NMR equipment (Bruker BioSpin). All signals were scaled to the intensity of the internal standard (TSP) of each sample, but no further scaling or normalization was performed. This meant that the results must be interpreted as absolute (quantitative) change but not as fold changes relative to a baseline level. An untargeted metabolomics approach was adopted, and it focused on broad identification of metabolites present in the samples. Spectral binning was performed using the package *mrbin* 1.7.4 (Klein, 2021) in R 4.3.0 and RStudio 2023.03.0 Build 386 (The R Foundation for Statistical Computing, Vienna, Austria). All spectra in the range of 0.5 ppm − 9.5 ppm were divided into equally sized segments (bins) of 0.005 ppm width and the noise and solvent signals were removed.

### Multivariate and statistical analysis

Principal Component Analysis (PCA) was performed to demonstrate variations between the samples and to identify outliers of concern. To visualize relationships between the samples and identify potential metabolite clusters, hierarchical clustering analyses were performed, and the results presented as heatmaps.

All linear models were created separately for each bin, and separately per germinant (TSB/AGFK), to screen for signals that significantly changed throughout the germination process according to the following formula:

1 $$Signal{\text{ }}intensity\,\sim \,Time{\text{ }}\left[ {hours} \right]$$

The p-values were calculated for each linear model and corrected for multiple testing using False Discovery Rate (FDR) at the 20% level. Only spectral signals that increased over time were analyzed in order to focus on the small molecules that were released during the germination process. Molecules decreasing during this time might be attributable to consumption by fully germinated bacteria. A post-hoc means comparison was done using Tukey’s Multiple Comparison Test to compare the means of significant signals at each time point.

### Metabolite identification

All metabolites were identified by comparing their chemical shifts signals of interest with the Human Metabolome Database (HMDB) (Wishart et al., 2022), and the Biological Magnetic Resonance Data Bank (BMRB) (Hoch et al., 2023), aided by comparison with the recorded HSQC spectra.

##  Results

### Multivariate analysis

For the spores that germinated with either AGFK or TSB germinant, samples from the media were collected throughout the germination process and tested by NMR to identify metabolite changes that occurred. A total of 36 samples were tested and PCA plots were created for each germinant. These PCA plots, including those from the media control samples, are shown in Supplemental Figures S1 and S2.

**Fig. 1:**
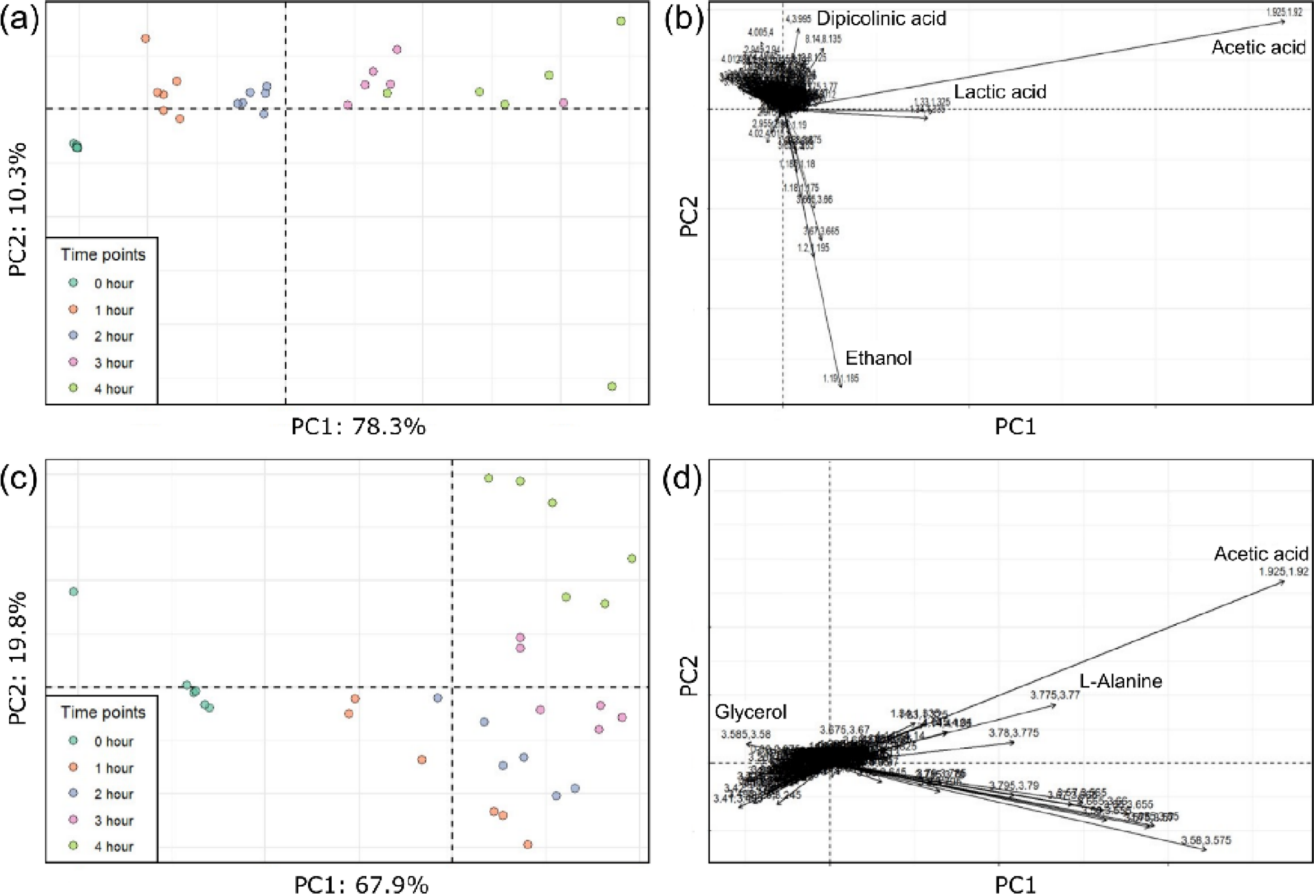
The PCA of the spores’ germination in different germinant solutions. (a) AGFK scores plot; (b) AGFK loadings plot showing features of importance; (c) TSB scores plot; (d) TSB loadings plot showing features of importance

In the PCA for the AGFK germinant (Fig. 1a), the samples collected at different time points show clear separation along PC1, corresponding to progressive germination. The associated loadings plot (Fig. 1b) shows signals contributing to this separation, with acetate, lactate, and dipicolinic acid being the main contributors to the separation along PC1, while ethanol contributed to the separation along PC2, with one sample at the 4-hour time point exhibiting unusually high ethanol levels. A similar pattern is observed for the TSB-induced germination (Fig. 1c). In this case, the samples separated along PC2 during the late germination period (4 h time point). For the TSB results in Fig. 1d, acetate, glycerol, and L-alanine were tentatively identified as primary metabolites driving this temporal separation. Of note, NMR does not distinguish between an acid and its salt, therefore it is common practice to use terms such as “acetate” and “acetic acid” interchangeably when referring to NMR results.

### Univariate analysis

**Fig. 2:**
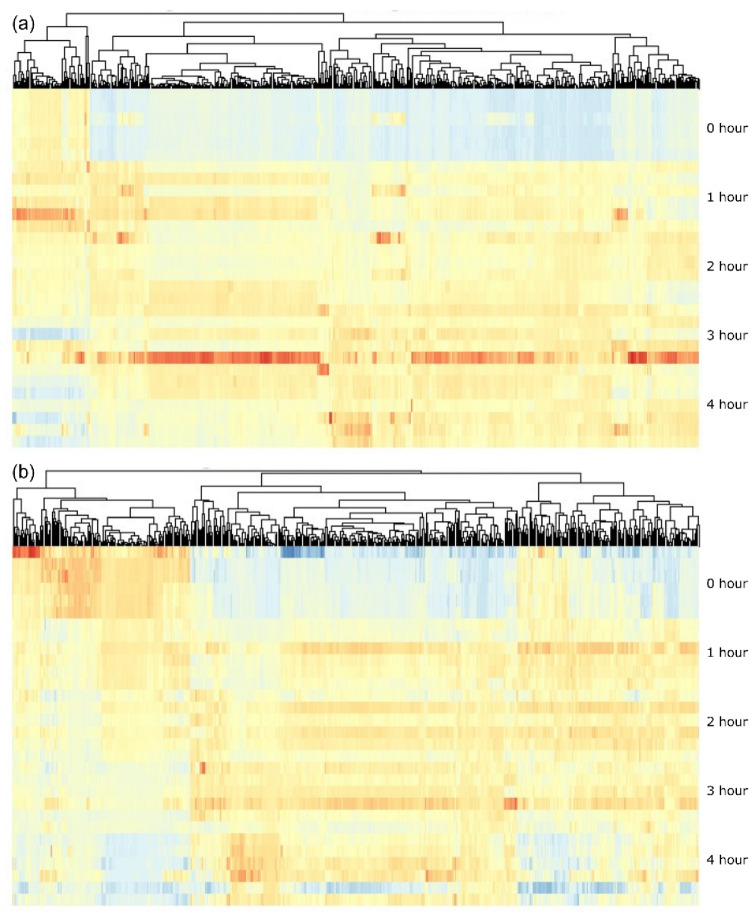
Heatmaps of significant features in AGFK-induced spores’ germination (a) and TSB-induced spores’ germination (b). x-axis: Significant features, y-axis: time points. Red: High abundance, yellow: medium abundance, blue: low abundance

Linear modeling was employed to assess spectral features that significantly changed during the spore germination process. The distribution of p-values was heavily skewed toward the left in both AGFK and TSB, hinting at true changes in the metabolic profiles during the germination of the spores (Supplemental Fig. S3). A False Discovery Rate (FDR) analysis revealed that 643 signals significantly changed in the AGFK group and 698 signals changed in the TSB group. The heatmaps (Fig. 2) show the abundances and distributions of the significant signals over time during the AGFK (Fig. 2a) and TSB-induced (Fig. 2b) spores’ germinations.

After excluding the signals that decreased during the spores’ germination, 7 metabolites were identified in AGFK-induced germination, while 5 were identified in the TSB group. The metabolite identified and matching chemical shifts are shown in the Supplemental Table S1, and additional information regarding metabolite identification can be found in Supplemental Fig. S4-S15.

### Time-dependent behavior

**Fig. 3:**
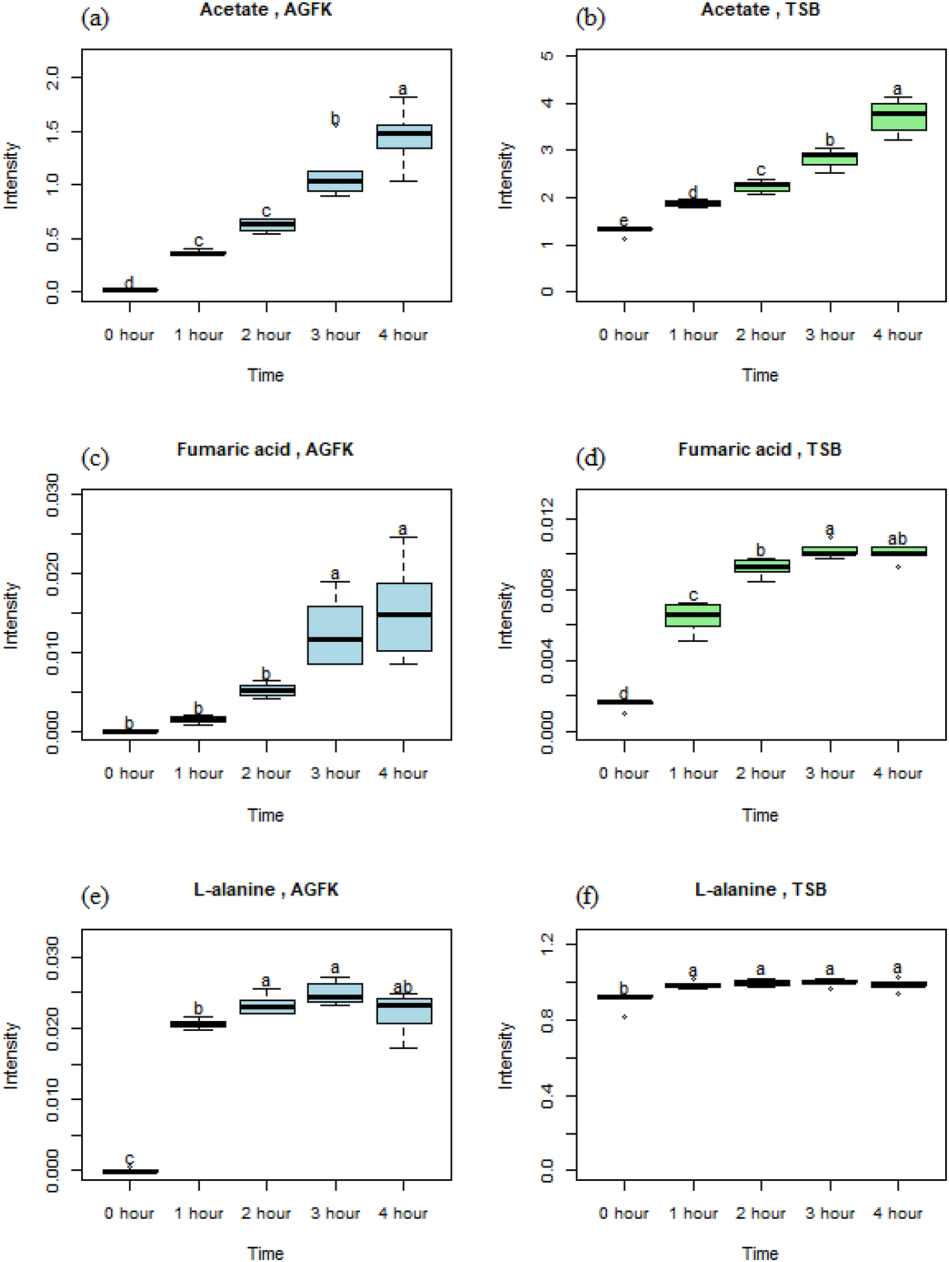

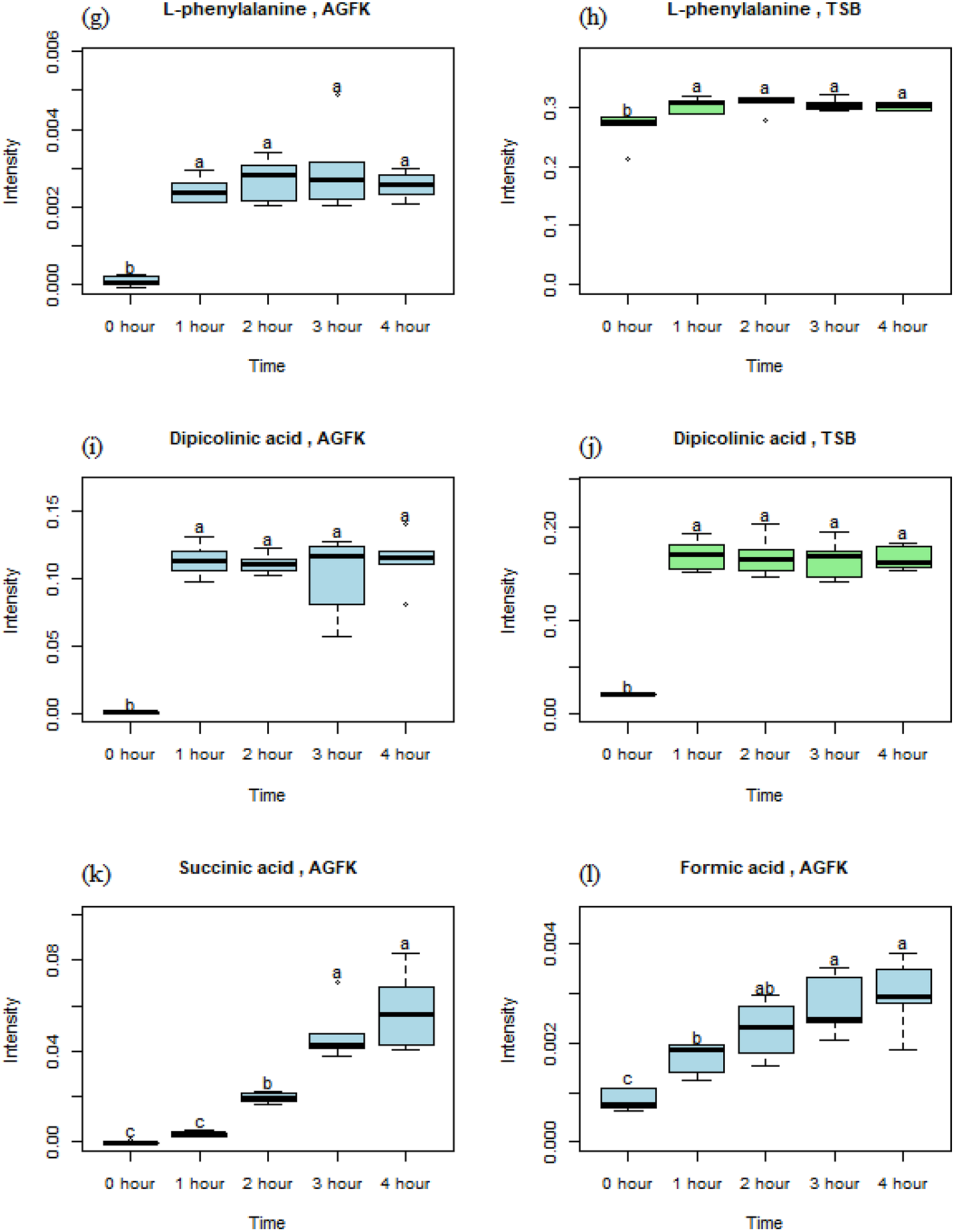
Metabolites generated during the spores’ germination. Differential letters indicate significant differences between time points. (a) acetic acid/AGFK, (b) acetic acid/TSB, (c) fumaric acid/AGFK, (d) fumaric acid/TSB, (e) L-alanine/AGFK, (f) L-alanine/TSB, (g) L-phenylalanine/AGFK, (h) L-phenylalanine/TSB, (i) dipicolinic acid (DPA)/AGFK, (j) DPA/TSB, (k) succinic acid/AGFK, (l) formic acid/AGFK

Figure 3 shows visualizations of significant metabolites during the spores’ germination with TSB and AGFK. Acetic acid emerged as a prominent metabolite with near-linear increasing abundance over time in both germinants, with TSB yielding a higher final concentration when compared to the AGFK (Fig. 3a, b). However, the elevated signal intensity at the 0-hour time point hints at the presence of acetic acid in TSB, even before the germination process began. Fumaric acid also exhibited a time-dependent increase, with a stronger presence observed in the AGFK-induced germination (Fig. 3c, d). L-alanine exhibited distinct trends, and it seems to depend on the germination media. In TSB (Fig. 3e), its signal intensity initially increased but declined slightly at the 4-hour time point. In AGFK (Fig. 3f), it displayed a steady rise throughout the spores’ germination. L-phenylalanine and dipicolinic acid displayed similar behavior in both TSB and AGFK (Fig. 3g-j). Their abundances increased over time, reaching peaks at 3 h before showing a slight decrease at the 4-hour time point. For formic acid, it started at a low level but showed a gradual increase during the AGFK-induced germination (Fig. 3k). Succinic acid followed a similar pattern, starting from zero intensity and accumulating at a higher level over time (Fig. 3l).

## Discussion

The growth medium was collected at various time points (0–4 h) to monitor the time-dependent dynamics of the metabolites released by the germinating spores. A total of 36 samples (6 replicates for each time point) were collected for each germination experiment (TSB and AGFK). A 4-hour germination period was chosen to ensure that > 90% of spores germinated within the timeframe. This was verified using a phase contrast microscopy, as described by Kong et al. (2011). To ensure optimal germination, a consistent pH of 7.0 was maintained for both nutrient solutions, since the germination of spores is effective at neutral pH, and most enzymes exhibit high activity at a pH range of 7–8. Tryptic soy broth (TSB) is a rich medium that contains proteins. To prevent protein interference with the quality of the NMR data, the samples were ultrafiltered to remove high molecular weight molecules (> 10 kDa). Additionally, media with ungerminated spores were collected and used as control samples (0-hour). The NMR spectroscopy method was chosen due to its robustness, its ability to identify various classes of molecules, and its high reproducibility (Guennec et al., 2014; Brennan, 2014).

### Principal component analysis (PCA)

Time-dependent trends in metabolic profiles during the AGFK- and TSB-induced spore germination are clearly visible in the PCA plots (Fig. 1). This figure shows that the AGFK samples exhibited separation along the PC1 axis, accounting for 78.3% of the variance. A progressive shift from left to right was observed, with 0-hour samples (pre-germination) positioned at the far left and 4-hour samples at the far right, indicating a gradual divergence in metabolic profiles over time. Partial overlap between the 3- and 4-hour samples suggests a higher metabolic similarity between these late-stage parts of the germination process. In Fig. 1c (TSB), a similar separation pattern was noted, with 67.9% variance explained along PC1. Here too, 0-hour and 4-hour samples marked the extremes along PC1, though the 4-hour samples also shifted slightly upward, suggesting a non-linear trajectory of metabolic changes. The 0-hour samples formed a distinct cluster, and no overlap was observed between the time points, underscoring the clear temporal progression in metabolic signatures during the germination process.

### Linear models

The heatmap visualization of linear model analysis (Fig. 2a, b) highlights dynamic metabolite abundance changes during the spores’ germination. In both germinants, the 0-hour replicates were dominated by blue regions, indicating low metabolite levels associated with the dormant spore state. At the 3-hour time point for the AGFK samples, a surge in metabolite production was observed, marked by red regions, followed by shifts, suggesting metabolic reprogramming. The late-stage parts of the germination (3–4 h) showed heterogeneity, with a mix of yellow and red regions, indicating increased metabolite abundance. With the TSB germination, similar patterns emerged, with the blue regions persisting in some of the 4-hour replicates, reflecting variability across the samples. Unexpected blue regions in the TSB germination suggest that some metabolites returned to low levels, while others increased significantly during the late germination stages, warranting further investigation.

### Identified metabolites

The metabolites that were identified could be broadly classified into two groups, based on temporal patterns. These were the metabolites that were clearly linked to early germination-specific events, including DPA, L-alanine, and L-phenylalanine, and those that showed metabolic changes both during the early and late germination periods (succinic acid, formic acid, acetic acid). The latter group may have been influenced by the onset of vegetative growth in some of the cells after 3–4 h of incubation. Fumaric acid showed an intermediate pattern, especially in TSB, where its increase in intensity plateaued after 2–3 h.

#### Acetic acid

During *Bacillus subtilis* spore germination, acetic acid release varied depending on the germinant. The AGFK-induced germination (Fig. 3a) shows no detectable acetic acid at 0 h, with levels increasing to 1.5 arbitrary units by the 4-hour time point. In the TSB-induced germination (Fig. 3b), acetic acid levels rose from an initial 1.3 units at 0 h to 3. 8 at the 4-hour time point, indicating a higher amount of release. Statistical analyses confirmed that significant changes in acetic acid levels occurred across the time points by both germinants, except between the 1 st and 2nd hours in AGFK, suggesting a potential lag phase. Acetic acid is a common metabolite generated by various pathways, including glycolysis and the TCA cycle (De Mey et al., 2007; Millard et al., 2021). The activation of these pathways during germination, as the dormant spore transitions to a metabolically active state, may be a source of acetic acid production, as reported by Zhuang et al. (2019). Another potential contributor to the observed increase could originate from spore amino acid metabolism, consistent with findings by Setlow et al. (1977), who demonstrated that acetic acid production by *Bacillus megaterium* spores, even in the absence of external carbon sources, suggested the utilization of internal reserves such as amino acids. Furthermore, acetic acid might play a role in regulating the internal and external pH of the environment of the germinated spores (Trunet et al., 2024). These pH changes may well influence the enzymatic activities during the revival of the spores, where acetic acid could act as a modulator, potentially influencing the reaction rates and overall metabolic flux. Future investigations into specific interactions between acetic acid and the key enzymes or regulatory pathways during germination would be valuable for the elucidation of its precise role in this critical life stage for the *Bacillus subtilis* spores.

#### Fumaric acid

The AGFK-induced germination (Fig. 3c) shows no detectable fumaric acid at 0 h, with a gradual increase over time. In TSB-induced germination (Fig. 3d), fumaric acid levels show an early surge, with a significant increase from 0 to 1 h, and leveling off to a plateau from 2 to 4 h. This suggested a rapid initial metabolite response. Despite the different temporal dynamics, both conditions reached similar maximum levels. The identification of fumaric acid during germination is intriguing because it is an intermediate metabolite within the Tricarboxylic acid (TCA) cycle, a central pathway for energy production in bacteria (Choi et al., 2021). In the TCA cycle, fumaric acid is converted into malic acid by the enzyme fumarase which helps in the generation of nicotinamide adenine dinucleotide + hydrogen (NADH) and flavin adenine dinucleotide, reduced form (FADH_2_) used in oxidative phosphorylation to produce adenosine triphosphate (ATP) (Nolfi-Donegan et al., 2020). The observed increase in fumaric acid during germination coincided with the reactivation of metabolic pathways that occurred as dormant spores transitioned to vegetative cells. This suggests a possible link between fumaric acid production and the restarting of the TCA cycle during the spore germination. Fumaric acid is connected to amino acid metabolism. It can be derived from aspartate and can serve as a precursor for the synthesis of several amino acids which may serve as energy sources during outgrowth of the cells (Conway, 2021). Further investigation into the enzymatic activities and metabolite flux within the TCA cycle would be valuable to solidify this connection and elucidate the precise role of fumaric acid in this process.

#### L-alanine

The behavior of L-alanine is illustrated in Fig. 3e and f. L-alanine was absent at 0 h in the AGFK media, but increased from 1 h onward, showing a slight decline at the 4-hour time point, with 0 h being significantly different from all the later time points. In contrast, L-alanine levels were already high at 0 h in the TSB and gradually increased to peak at 3 h before slightly declining at 4 h, likely due to its metabolic utilization during outgrowth of the cells. Although the intensity varied over time, no significant differences were observed between the 1- to 4-hour time points. The source of the released L-alanine may be the degradation of small acid soluble proteins (SASPs) in the core of the spores (Vyas et al., 2011). Vyas et., (2011) also reported that this degradation released free amino acid and occurred after the hydrolysis of the cortex. This release of L-alanine during the *Bacillus subtilis* spore germination in our study, aligns with the results reported by Dodatko et al. (2009). Their findings reported the release of 7 amino acids, including L-alanine, during the germination of *Bacillus cereus* spores that were triggered by inosine, despite L-alanine not being present in the spores. The phylogenic relationship between *Bacillus subtilis* and *Bacillus cereus* suggests that this might be a conserved metabolic response to germination. Setlow et al. (2008) observed the release of free amino acids during germination of *Bacillus subtilis* and *Bacillus megaterium* spores, and they attributed this to the hydrolysis of small acid-soluble spore proteins (SASPs) as reported by Tovar-Rojo et al. (2003). However, the specific amino acids reported in their study were arginine and glutamic acid. Our finding of L-alanine release thus contradicts the results of Setlow et al. (2008). The differences in the amino acids released could be due to the different bacterial strains involved.

The reduction in L-alanine levels at the 4-hour time point suggests that this amino acid also served as a readily available nutrient that was utilized by the germinated spores. During outgrowth, the spores began to metabolize available nutrients to support growth and cell division (Chirakkal et al., 2002). As a simple amino acid, L-alanine can be deaminated to pyruvate, which then enters central metabolic pathways such as glycolysis and the TCA cycle, providing energy and metabolic intermediates necessary for cell growth (Dave & Kadeppagari, 2019). Additionally, this amino acid could serve as building blocks for the synthesis of proteins as the germinated spores resume active protein production (Sinai et al., 2015).

#### L-phenylalanine

In AGFK germination (Fig. 3g), L-phenylalanine was initially absent but showed a sharp increase at the 1-hour time, remaining stable thereafter, with no significant differences across the later time points. In TSB germination (Fig. 3h), the signal intensity gradually increased between the 0- to 2-hour time points, followed by a decline at the 3- and 4-hour time points, possibly due to its metabolic utilization during the spores’ outgrowth. No significant differences were observed during the 1-, 2-, 3-, and 4-hour time points. In studies with spores, Vyas et al. (2011) reported that L-phenylalanine release occurred from the degradation of SASPs in the core of the spores. Unlike L-alanine’s direct entry into energy production, L-phenylalanine may function as a precursor in the biosynthetic pathway of various secondary metabolites (Reshi et al., 2023). These secondary metabolites, including phenylpyruvate and other aromatic compounds, could play diverse roles within germinating spores. Phenylpyruvate, for instance, could potentially enter the TCA cycle, contributing to ATP production which is needed by the germinated spores for other cellular activities. Additionally, L-phenylalanine possesses the potential to be converted into tyrosine and tryptophan through enzymatic pathways (Wiggins et al., 2015). Both tyrosine and tryptophan are essential amino acids that are involved in a multitude of cellular functions, including protein synthesis and signal transduction which are needed during the outgrowth of germinating spores.

#### Dipicolinic acid (DPA)

This is a major constituent of the *Bacillus subtilis* spore core. It plays a critical role in maintaining dormancy by chelating calcium ions (Ca²⁺), which in turn binds water molecules and promotes dehydration of the spore’s cores (Lyu et al., 2023). This dehydration state suppresses enzymatic activity and thus preserves the spore’s dormancy. As shown in Fig. 3i, during AGFK-induced germination, DPA was completely absent at the 0-hour time point, highlighting its retention within the dormant spore. A sharp increase in signal intensity at the 1-hour time point indicated rapid DPA release, and this signaled the initiation of germination. From the 1- to 4-hour time points, the levels remained statistically unchanged, suggesting that the majority of DPA was released early in the process. A similar pattern was observed in TSB-induced germination (Fig. 3j), where DPA was also absent at the 0-hour time point, but rose sharply by the 1-hour time point, and then plateaued, with no significant change in intensity between 1 and 4 h. This consistent early release pattern highlighted DPA’s role in initiating germination through core rehydration. These findings align with previous studies, including those from Porębska et al. (2016), which described DPA release as essential for spore core hydration. Research by Francis and Sorg (2016) and Kong et al. (2011) further supports this mechanism, linking DPA or CaDPA release to both biochemical signaling and the loss of phase-brightness observed in germinating spores. Together, these results confirm that DPA release is a key biochemical event that facilitates the transition from spore dormancy to active vegetative growth.

#### Succinic acid

This is a key intermediate in the TCA cycle and was only significant in the AGFK-induced spore germination, suggesting its production was linked to the reactivation of energy-generating pathways as the spores transitioned to vegetative cells. As shown in Fig. 3k, succinic acid was absent at the 0-hour time point but gradually increased over time, peaking at a signal intensity of 0.05 after 4 h. Although the levels at 0 and 1 h were statistically similar, they differed significantly during later time points, while 3- and 4-hour levels were also similar, but distinct from earlier stages. This trend highlighted a time-dependent activation of central metabolism, with succinic acid potentially serving as an energy source for the spores’ outgrowth. Further research is needed to clarify its precise role during germination.

#### Formic acid

This showed a significant increase only during AGFK-induced spore germination, as depicted in Fig. 3l. Its gradual rise from the 0-hour time point, though produced in small quantities (maximum intensity ~ 0.003 arbitrary units), suggests involvement in early metabolic reactivation. Its initial presence may have stemmed from amino acid degradation, likely released from the breakdown of SASP in the spore core (Vyas et al., 2011). As germination progressed, formic acid may have oxidized to carbon dioxide, contributing to NADH generation and fueling the TCA cycle for ATP production. These steps are critical for spores outgrowth and the proliferation of vegetative cells (Alothaim, 2023). Additionally, formic acid may serve as a precursor for nucleotide and amino acid biosynthesis, supporting DNA, RNA, and protein syntheses that are required for cell growth and division (Oizel et al., 2020; Kim et al., 2019).

### Limitations

This study has several limitations: First, quantitative growth measurements such as optical density (OD) or colony forming units (CFU) could have provided further confirmation of growth phase transitions in addition to the phase contrast microscopic observations. Second, due to the complex composition of TSB, thermal degradation may contribute to the observed change in metabolic composition throughout the growth experiment. However, for most identified metabolites, metabolic changes occurred at almost identical rates in both TSB and AGFK. This highlights that these specific effects cannot be driven by large-scale degradation of TSB constituents. Still, thermal degradation will occur to some extent, and analysis of pure (spore-free) media samples after incubation could have provided us with information of intrinsic temporal changes occurring in the growth environment. Additionally, TSB is a complex mixture and, thus, more susceptible to peak overlap in NMR experiments. The use of a high field magnet at 850 MHz reduced the issue of peak overlap, and we confirmed the identities of all metabolites discussed in this paper by comparisons of chemical shifts, multiplet patterns, coupling constants (“tentative match”), and/or positional matches in HSQC spectra. These constitute the gold standard of metabolite identification (“confirmed match”). Visibly overlapped peaks were not used for identification purposes. Still, residual peak overlap might have obscured other metabolites that might have also shown significant changes, leaving gaps in our analysis. In future studies we will address this issue.

Another potentially confounding factor is the fact that, once the spores have outgrown into vegetative cells, they will exhibit regular metabolic activity, which could confound the metabolic changes caused during germination. Microscopic analyses performed during the method development showed that significant numbers of rod-shaped vegetative cells were only visible after 5 h of growth. In addition, the fact that we saw significant metabolic changes in all the metabolites at the 1-hour time point, gave us confidence that these changes were due to germination, and not purely due to later outgrowth. Still, future studies should attempt to arrest the outgrowth by limiting the available nutrients or adding inhibitors to further disentangle these effects. Future studies should address these issues and also consider the use of an orthogonal technology such as liquid chromatography-mass spectrometry (LC-MS) that could further validate NMR analytical methods and provide information on low-abundance metabolites.

## Conclusions

This study identified metabolites released during chemically induced spore germination, and this provided valuable insights into microbial behavior that could be applicable to various fields such as agriculture, food, medicine and pharmaceutical disciplines. This study demonstrated a consistent metabolic profile in *Bacillus subtilis* OSU 494 spore germination in both AGFK and TSB media. This may indicate that this organism has a core set of metabolic pathways during germination, regardless of the nutrient germinant. Key metabolites such as acetic acid, fumaric acid, L-alanine, L-phenylalanine, DPA, succinic acid, and formic acid emerged as potential biomarkers for tracking the germination of bacterial spores. These findings have important implications, particularly for the food industry. While this study was done in buffer systems that simulated foods, more research is needed to understand such interactions in consumer products. Also, the present study evaluated only one strain of spore-forming bacteria, and further studies will be needed to validate how well the results can be generalized.

Food manufacturers could use biomarkers of spore germination to develop detection methods for *Bacillus subtilis* spores, enabling timely intervention strategies to prevent spoilage and ensure food safety. By linking specific metabolites to spore activation, this research demonstrated a potential method that supports advancements in targeted food safety approaches.

## Supplementary Material

Supplementary Material 1.

## Data Availability

The datasets used for the analysis in the current study are available from the corresponding author on reasonable request.
