# Supplementary material for: Nutrient-induced germination of Bacillus subtilis spores exhibiting shared metabolic profiles in both TSB and AGFK media
Source: Metabolomics. 2026 May 16;22(3):72. doi: 10.1007/s11306-026-02442-4 (PMC13179926; doi:10.1007/s11306-026-02442-4)
Supplement: Supplementary file 1 — Supplementary Material 1. [file 11306_2026_2442_MOESM1_ESM.docx]

**Nutrient-Induced Germination of *Bacillus subtilis* Spores Exhibiting Shared Metabolic Profiles in Both TSB and AGFK Media**

Nana Asiamah Boateng^1^, V. M. Balasubramaniam^1^, Melvin A. Pascall^1*^, Matthias S. Klein^2^

^1^Department of Food Science and Technology, The Ohio State University, 2015 Fyffe Road, Columbus, OH 43201, USA

^2^Department of Animal Science, McGill University, 21111 Lakeshore Road, Sainte-Anne-de-Bellevue, QC H9X3V9, Canada

* Correspondence: pascall.1@osu.edu, Phone: 614-292-0287, Fax: 614-292-0218

**SUPPLEMENTAL MATERIALS**

**Table S1**: Identified metabolite signals that significantly increased during spore germination.

| **Metabolite ID** | **Nutrient Germinant** | **Bin chemical shift (ppm)** | **Identification Confidence** |
| --- | --- | --- | --- |
| Acetic acid | AGFK | 1.93, 1.92 | Confirmed by HSQC |
|  | TSB | 1.93, 1.92 |  |
| Fumaric acid | AGFK | 6.53, 6.52 | Tentative (matches chemical shift & coupling pattern) |
|  | TSB | 6.53, 6.52 |  |
| L-alanine | AGFK | 3.78, 3.77;  1.49, 148;  1.48, 1.47 | Confirmed by HSQC |
|  | TSB | 3.78, 3.77;  1.49, 148;  1.48, 1.47 |  |
| L-phenylalanine | AGFK | 7.44, 7.43;  7.43, 7.42;  7.39, 7.38;  7.38, 7.37;  7.34, 7.33 | Confirmed by HSQC |
|  | TSB | 7.44, 7.43;  7.43, 7.42;  7.39, 7.38;  7.38, 7.37;  7.34, 7.33 |  |
| Dipicolinic acid | AGFK | 8.18, 8.17;  8.17, 8.16;  8.16, 8.15;  8.15, 8.14;  8.14, 8.13;  8.13, 8.12 | Tentative (matches chemical shift & coupling pattern) |
|  | TSB | 8.18, 8.17;  8.17, 8.16;  8.16, 8.15;  8.15, 8.14;  8.14, 8.13;  8.13, 8.12 |  |
| Succinic acid | AGFK | 2.41, 2. 4;  2.39, 2.38 | Confirmed by HSQC |
| Formic acid | AGFK | 8.47, 8.46;  8.46, 8.45 | Confirmed by HSQC |

Table S1 summarizes the identifications of metabolites that significantly changed, and whose abundances increased during the spores’ germination in TSB and/or AGFK germinants, indicating release rather than consumption. This table focuses specifically on metabolites exhibiting a time-dependent rise during 0 to 4 hours of germination. The table also includes the corresponding proton chemical shifts for each metabolite. Notably, the table includes a column titled "Identification Confidence" which indicates the level of confidence for each metabolite. Metabolites can be **tentatively** identified based solely on their proton chemical shifts, multiplicity patterns, and coupling constants, or **confirmed** by identifying matching carbon chemical shifts in the collected HSQC spectra. Interestingly, AGFK-induced germination yielded identifications for seven metabolites, while the TSB germination identified five. In both cases, two identifications were confirmed with HSQC, while the remaining metabolites received tentative assignments, mostly due to not being observable in the HSQC as a result of low abundance.


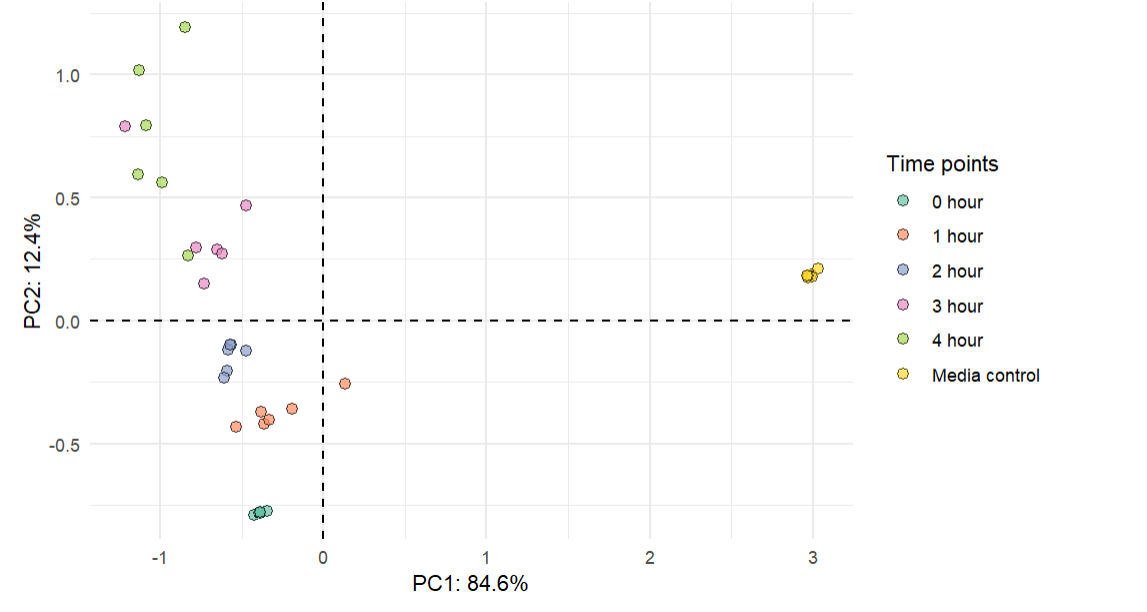


**Fig. S1:** PCA score plot showing the germination of spores in AGFK germinant at different time points including media controls.


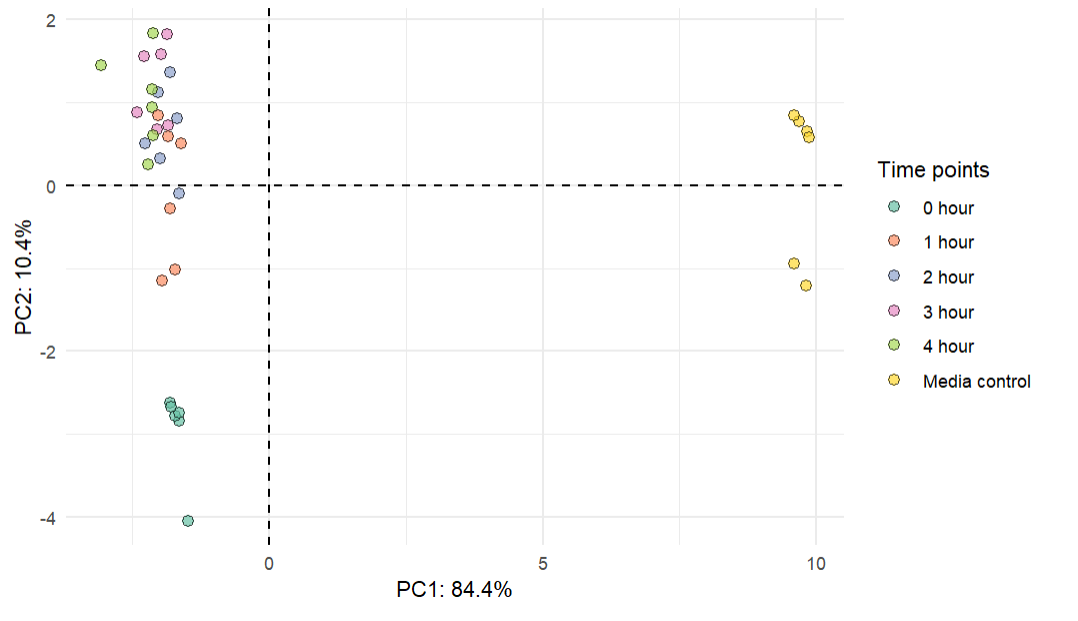
**Fig. S2**: PCA score plot of the spores’ germination in TSB germinant solution at different time points including media control.

The principal component analysis (PCA) revealed separations between the control media and the samples with AGFK and TSB induced spores’ germination. From Figures S1 and S2, there were wide separations between the media control and the samples collected at the timepoints (0 - 4 hours). This huge separation may have occurred because of the addition of the spores. The control media contained only the germinant solution without addition of the spores. In the PCA score plots, larger separations indicated greater dissimilarity between the samples. There was a separation between the samples collected at the timepoints along PC2. However, the smaller variance along PC2 indicated that the samples were not too different from each other since there were some overlapping of the samples collected from timepoints 1- 4 hours. This implied that the metabolic profiles during this active germination phase shared some characteristics. The high variance along PC1 shown in Figures (1a and 2a) might have masked more subtle separations between the samples collected at different germination time points (0 - 4 hours) along the PC2.


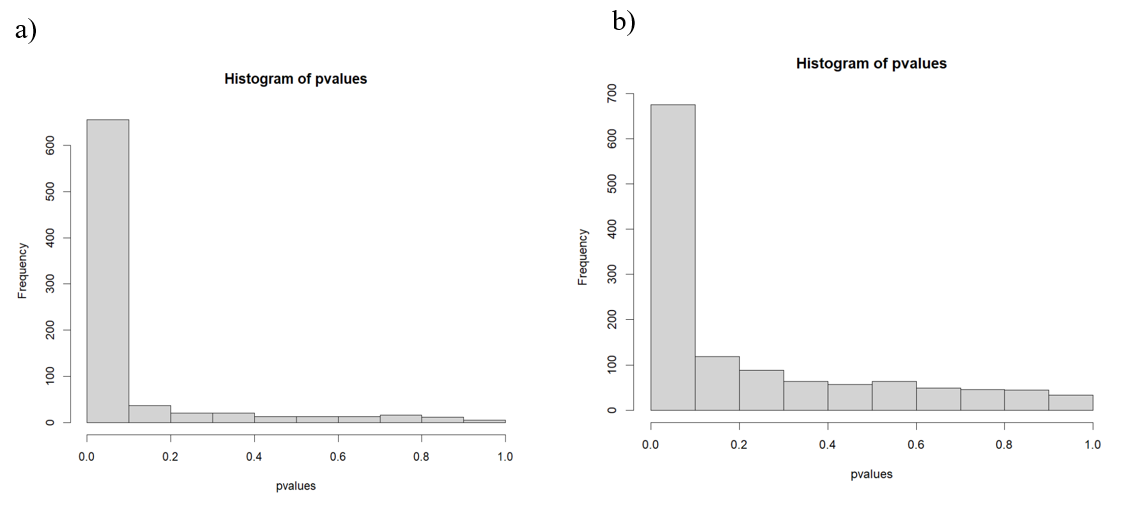


**Fig. S3:** Histograms of p-values from linear modelling of the spores’ germination at different time points using different germinant solutions. a) AGFK; b) TSB. The histograms reveal a distinct clustering of p-values near zero, indicating a strong enrichment for statistically significant changes in the metabolites’ abundances during the germination process. This pattern stands in contrast to a uniform distribution, which would signify random fluctuations rather than genuine alterations in the metabolic profiles.

a)


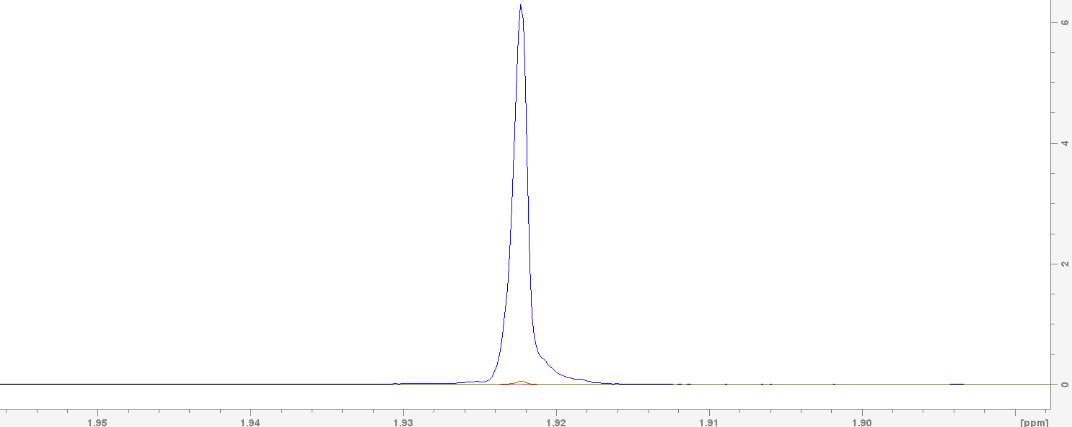


b)


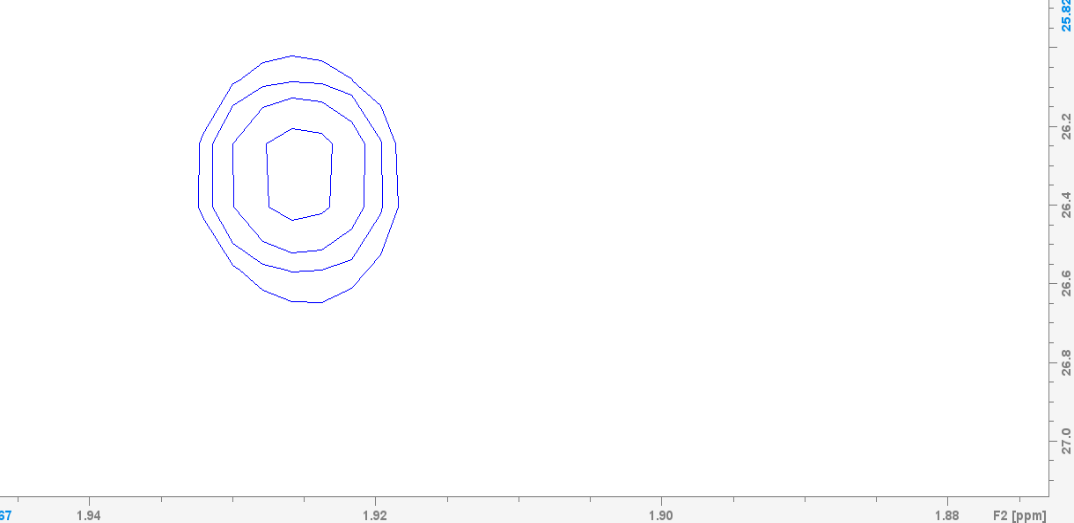


**Fig. S4**: a) 1D spectral overlay of acetate at 1.92 ppm produced by spores germinated in AGFK germinant solution. b) 2D signal of acetic acid at 1.92 ppm on the proton frequency and 26.5 ppm on the carbon frequency produced by spores germinated in AGFK germinant solution.

*Signals colored (green, red and blue) represent samples collected at; 0-hour time point (before germination), media control and 4-hour time point respectively.

a)


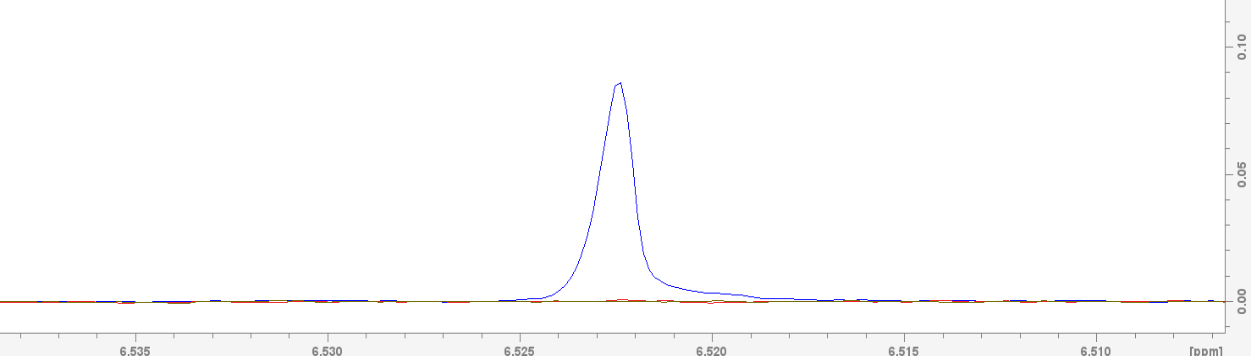


b)


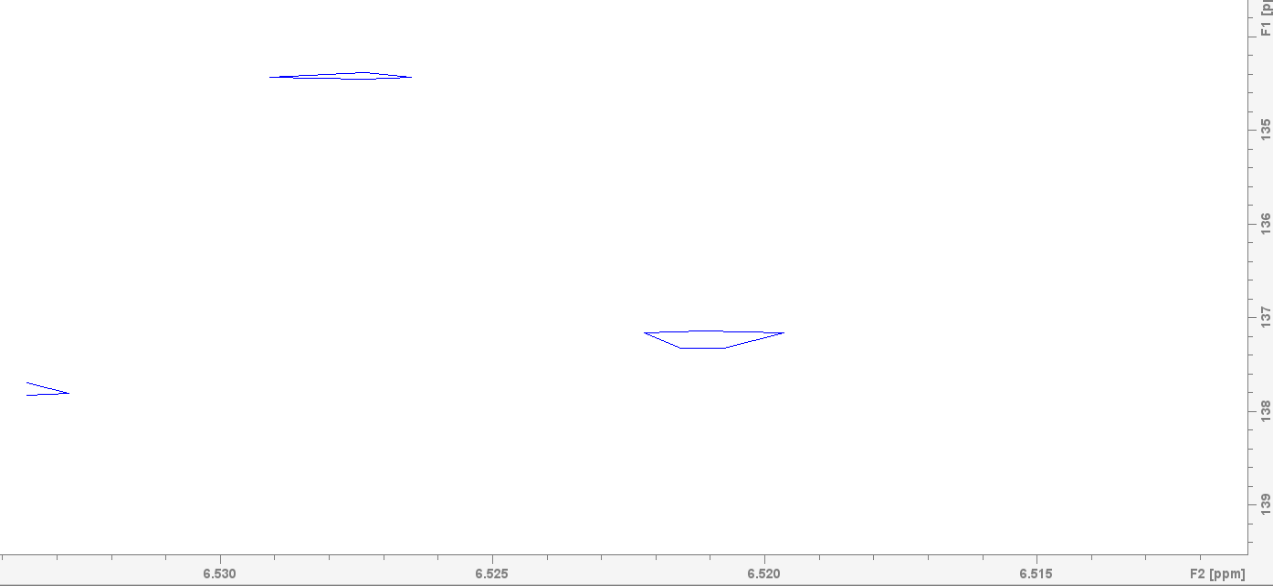


**Fig. S5**: a) 1D spectral overlay of fumaric acid at 6.52 ppm produced by spores germinated in the AGFK germinant solution. b) 2D signal of fumaric acid at 6.52 ppm on the proton frequency and 137 ppm on the carbon frequency produced by spores germinated in AGFK germinant solution.

*Signals colored (green, red and blue) represent samples collected at; 0-hour time point (before germination), media control and 4-hour time point respectively.


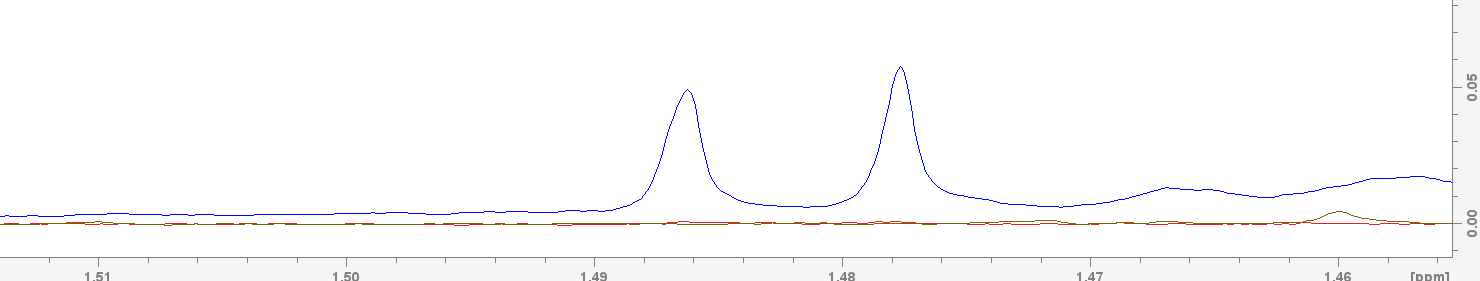


**Fig. S6**: 1D spectral overlay of L-alanine at 1.48 ppm produced from spores germinated with AGFK germinant solution.

*Signals colored (green, red and blue) represent samples collected at; 0-hour time point (before germination), media control and 4-hour time point respectively.

a)


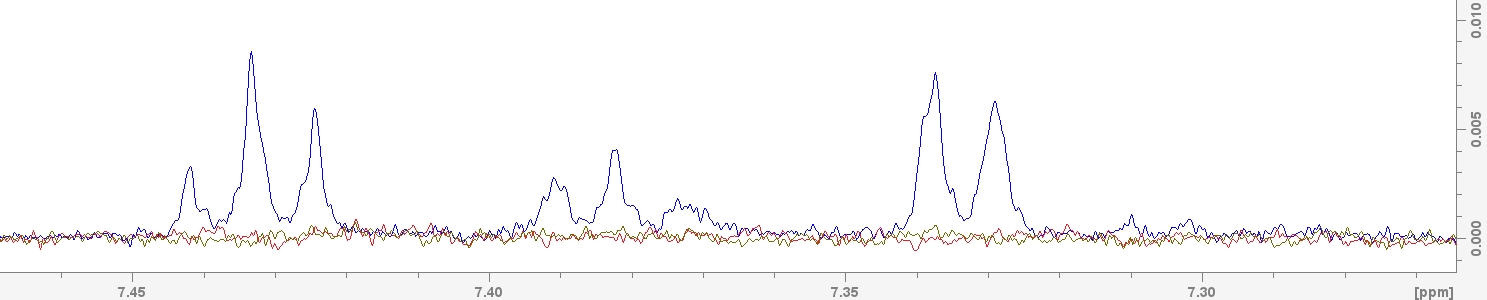


b)


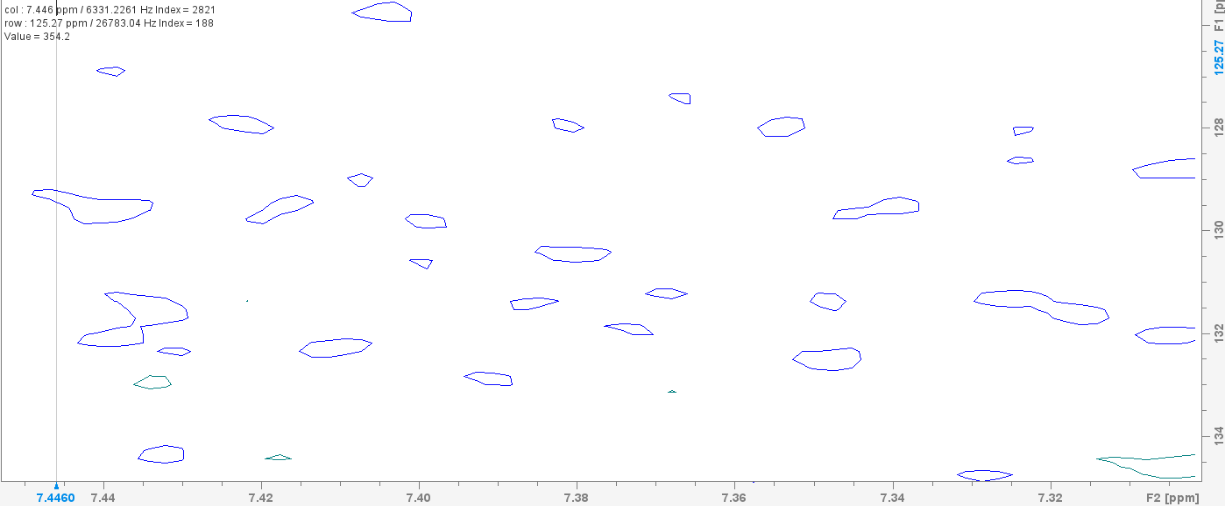


**Fig. S7**: a) 1D spectral overlay of L-phenylalanine at 7.44 - 7.42 ppm, 7.39 - 7.37 ppm and 7.34 - 7.33 ppm produced by spores germinated with AGFK germinant solution. b) 2D signal of L-phenylalanine at 7.44 - 7.42 ppm, 7.39 - 7.37 ppm and 7.34 - 7.33 ppm on the proton frequency and 131, 130 and 132 ppm on the carbon frequency produced by spores germinated in AGFK germinant solution.

*Signals colored (green, red and blue) represent samples collected at; 0- hour time point (before germination), media control and 4-hour time point respectively.

a)


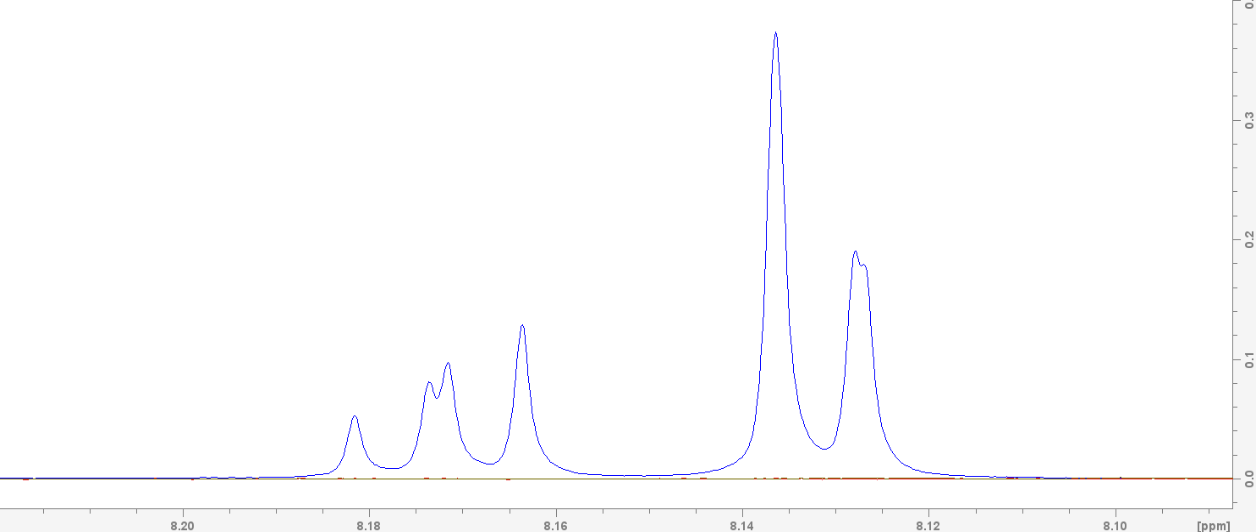


b)


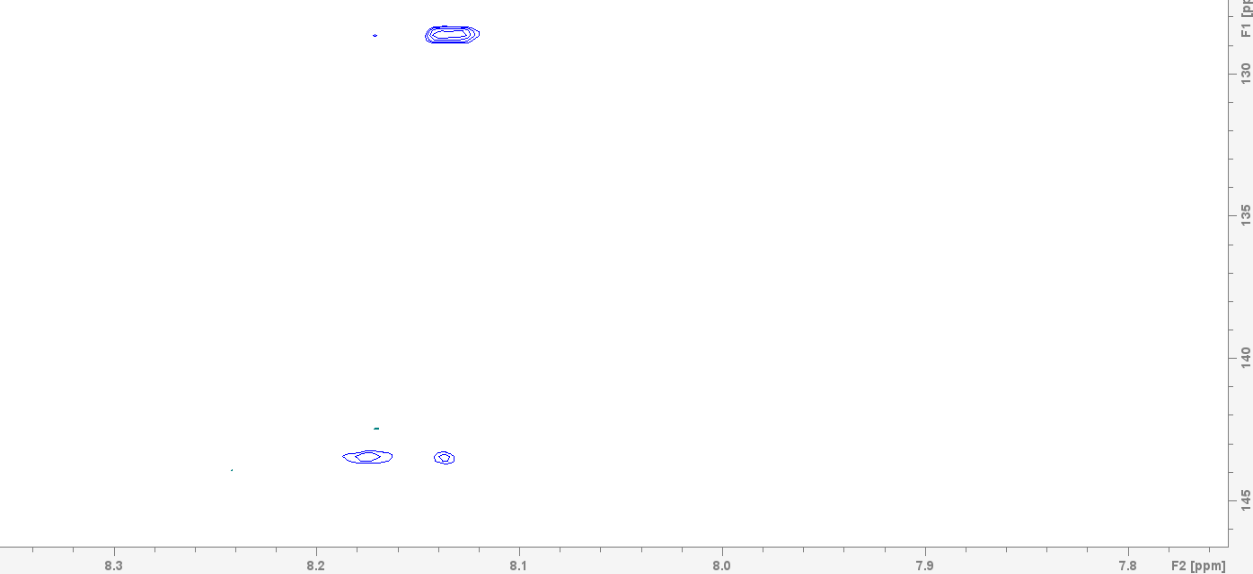


**Fig. S8**: a) 1D spectral overlay of dipicolinic acid at 8.18 - 8.12 ppm produced by spores germinated in AGFK germinant solution. b) 2D signal of dipicolinic acid at 8.18 - 8.12 ppm on the proton frequency and 129 and 143 ppm on the carbon frequency produced by spores germinated in AGFK germinant solution.

*Signals colored (green, red and blue) represent samples collected at; 0-hour time point (before germination), media control and 4-hour time point respectively.

a)


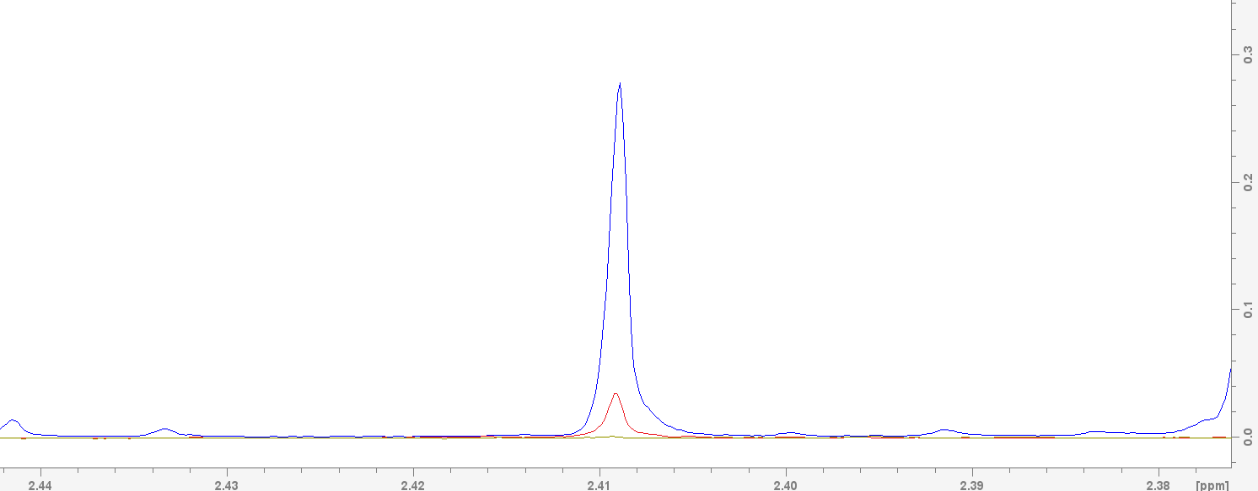


b)


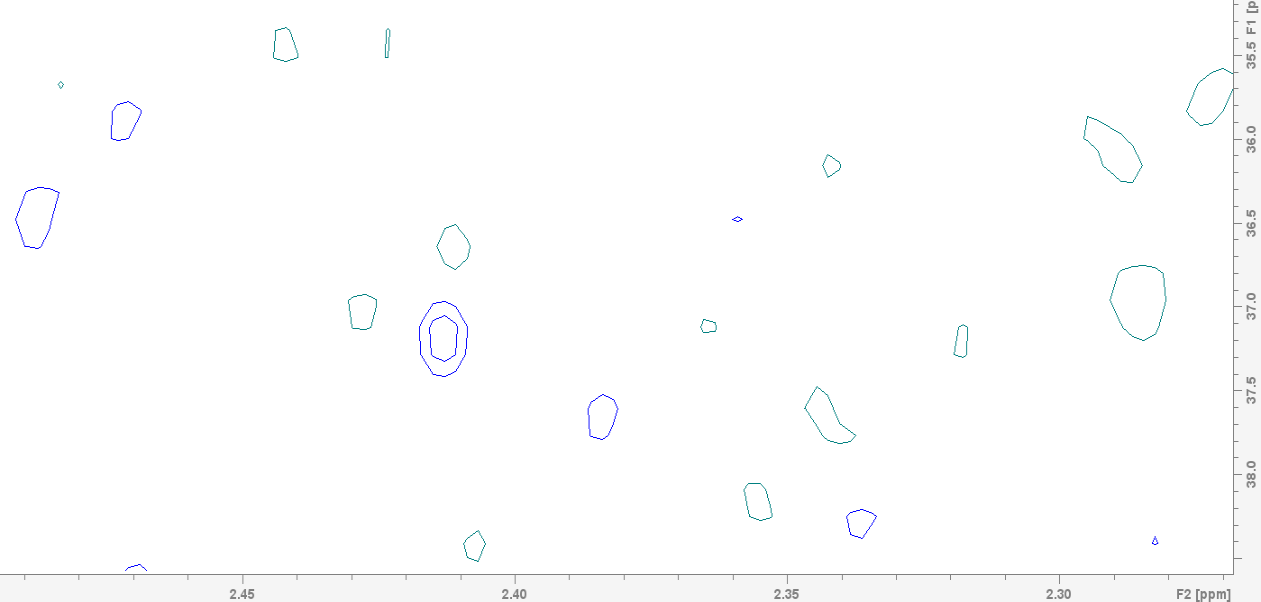


**Fig. S9**: a) 1D spectral overlay of Succinic acid at 2.41 ppm produced by spores germinated in AGFK germinant solution. b) 2D signal of Succinic acid at 2.41 ppm on the proton frequency and 37 ppm on the carbon frequency produced by spores germinated in AGFK germinant solution.

*Signals colored (green, red and blue) represent samples collected at; 0-hour time point hour (before germination), media control and 4-hour time point respectively.

a)


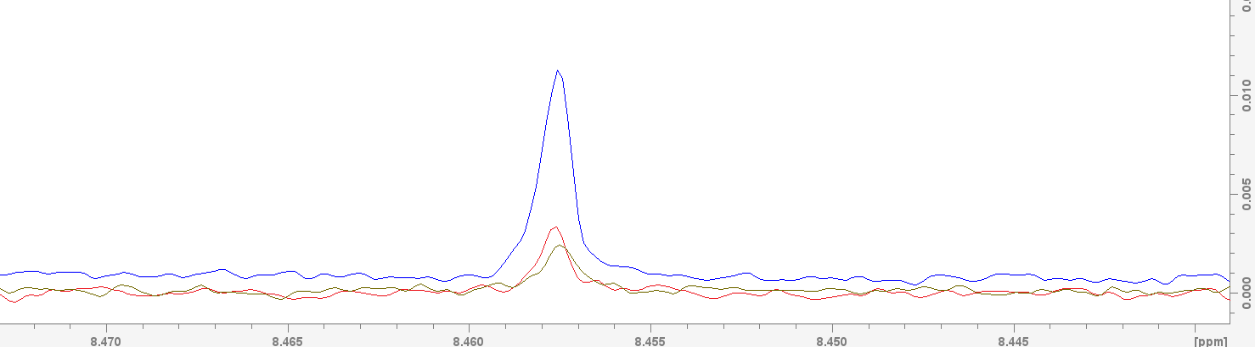


b)


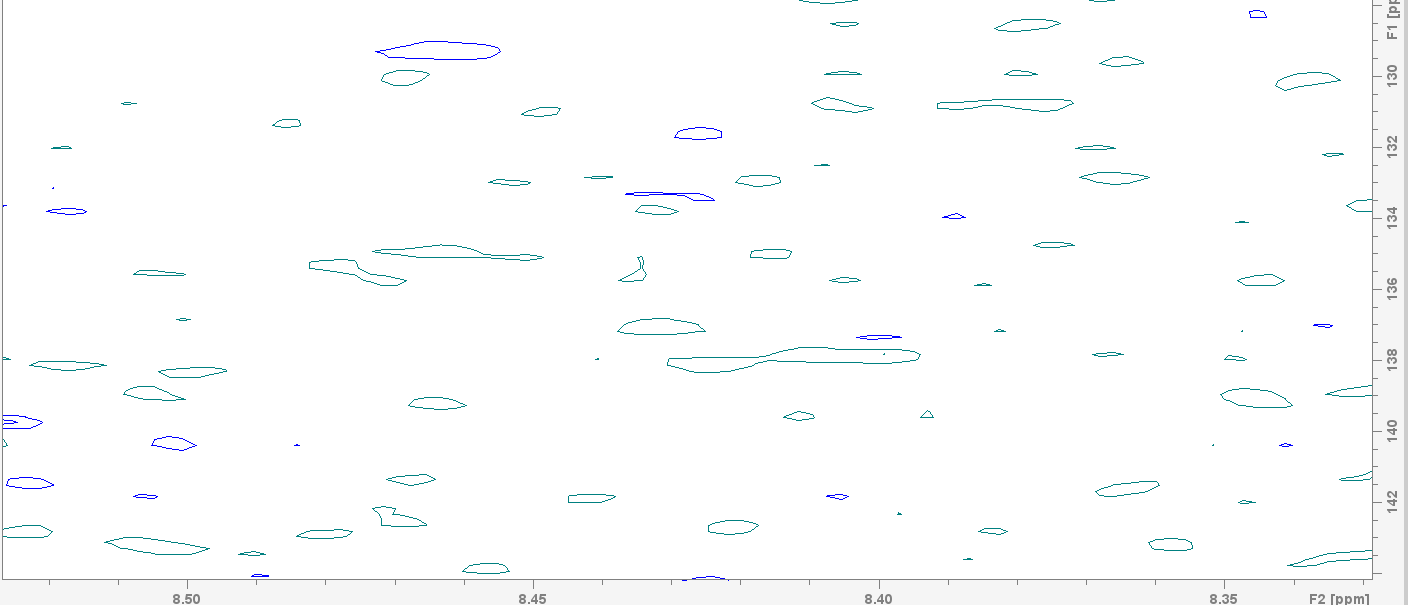


**Fig. S10**: a) 1D spectral overlay of Formic acid at 8.46 ppm produced by spores germinated in AGFK germinant solution. b) 2D signal of formic acid at 8.46 ppm on the proton frequency and 129 ppm on the carbon frequency produced by spores germinated in AGFK germinant solution.

*Signals colored (green, red and blue) represent samples collected at; 0-hour time point (before germination), media control and 4-hour time point respectively.

a)


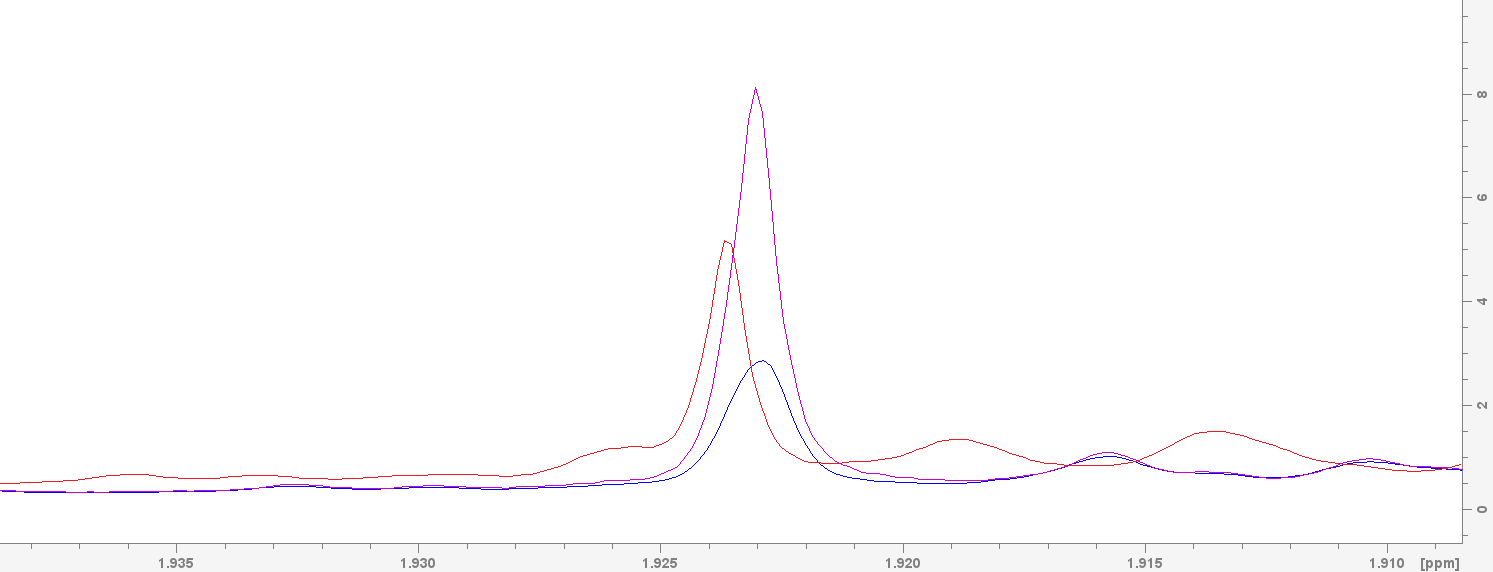


b)


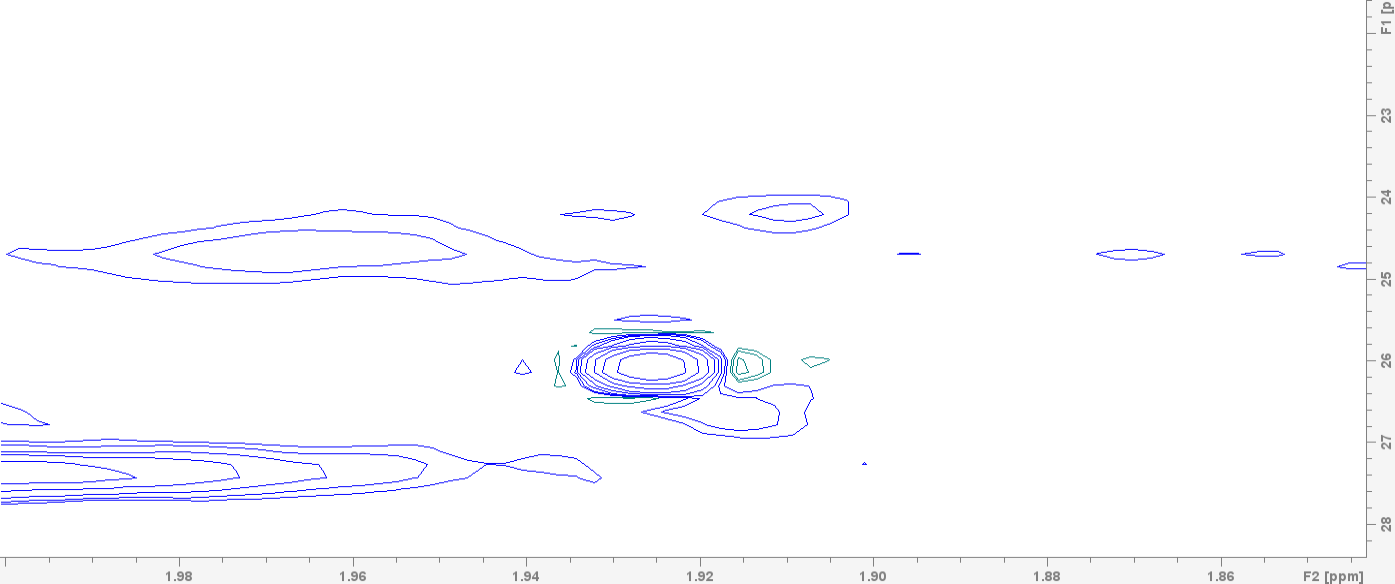


**Fig. S11**: a) 1D spectral overlay of signal of acetic acid at 1.92 ppm produced from spores germinated with TSB germinant. b) 2D signal of acetic acid at 1.92 ppm on the proton frequency and 26 ppm on the carbon frequency produced by spores germinated in TSB germinant solution.

*Signals colored (pink, red and blue) represent samples collected at; 3-hour time point, media control and 0-hour time point (before germination), respectively.

a)


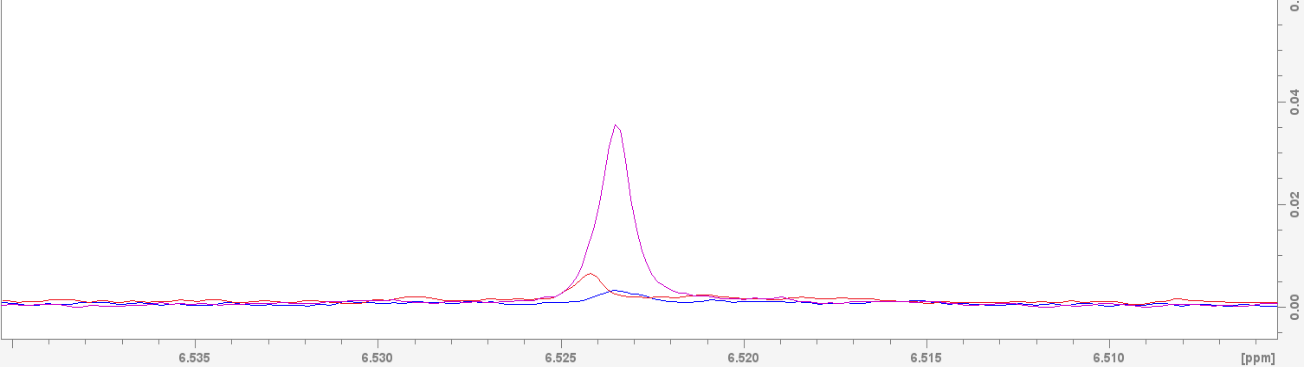


b)


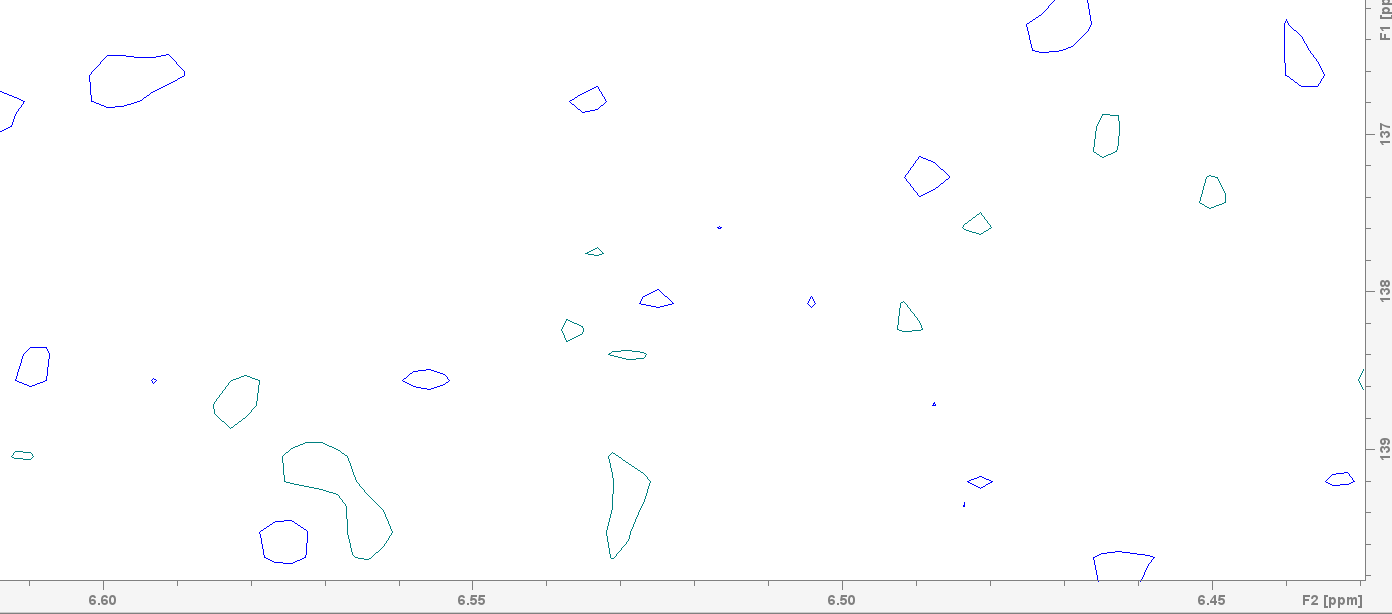


**Fig. S12**: a) 1D spectral overlay of signal of fumaric acid at 6.52 ppm produced from spores germinated with TSB germinant. b) 2D signal of fumaric acid at 6.52 ppm on the proton frequency and 137 ppm on the carbon frequency produced by spores germinated in TSB germinant solution.

*Signals colored (pink, red and blue) represent samples collected at; 3-hour time point, media control and 0-hour time point (before germination), respectively.

a)


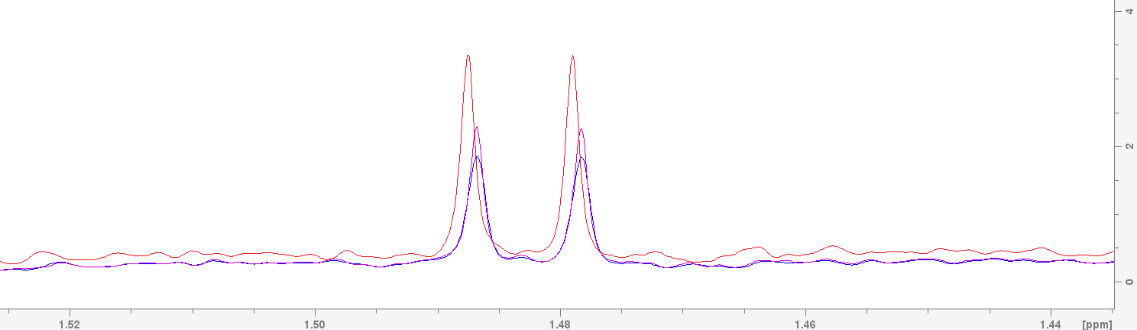


b)


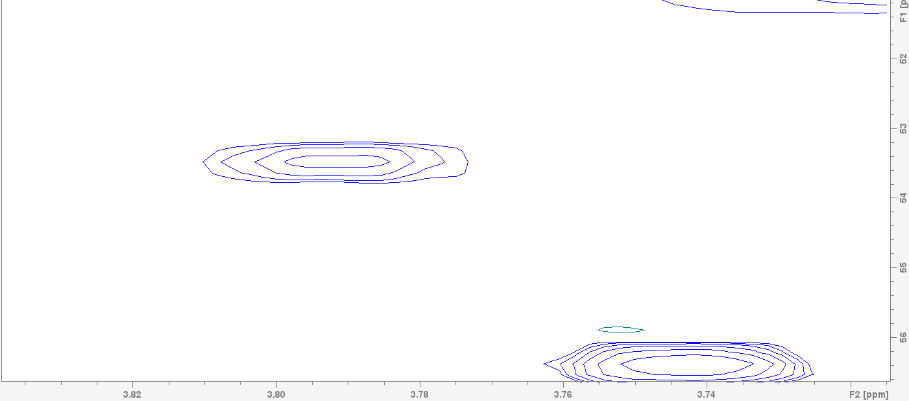


c)


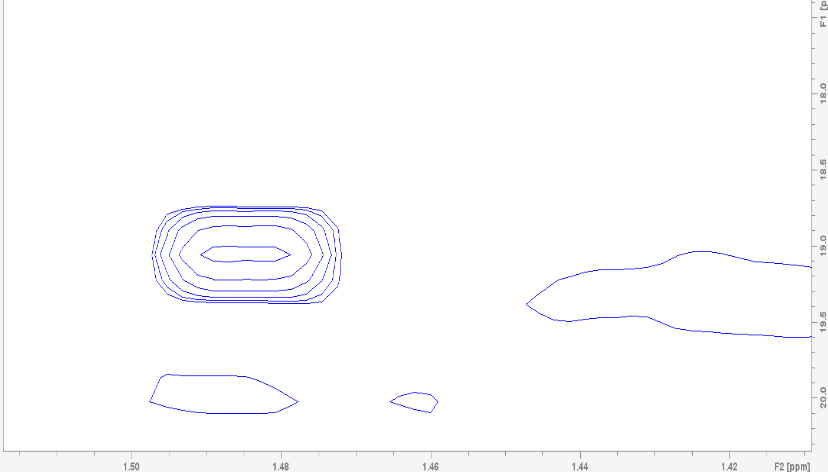


**Fig. S13**: a) 1D spectral overlay of L-alanine at 1.49 ppm produced by spores germinated with TSB germinant. b) 2D signals of L-alanine at 3.78 ppm on the proton frequency and 53 ppm on the carbon frequency produced by spores germinated with TSB germinant. c) 2D signals of L-alanine at 1.49 ppm on the proton frequency and 19 ppm on the carbon frequency produced by spores germinated with TSB germinant.

*Signals colored (pink, red and blue) represent samples collected at; 3-hour time point, media control and 0-hour time point (before germination), respectively.

a)


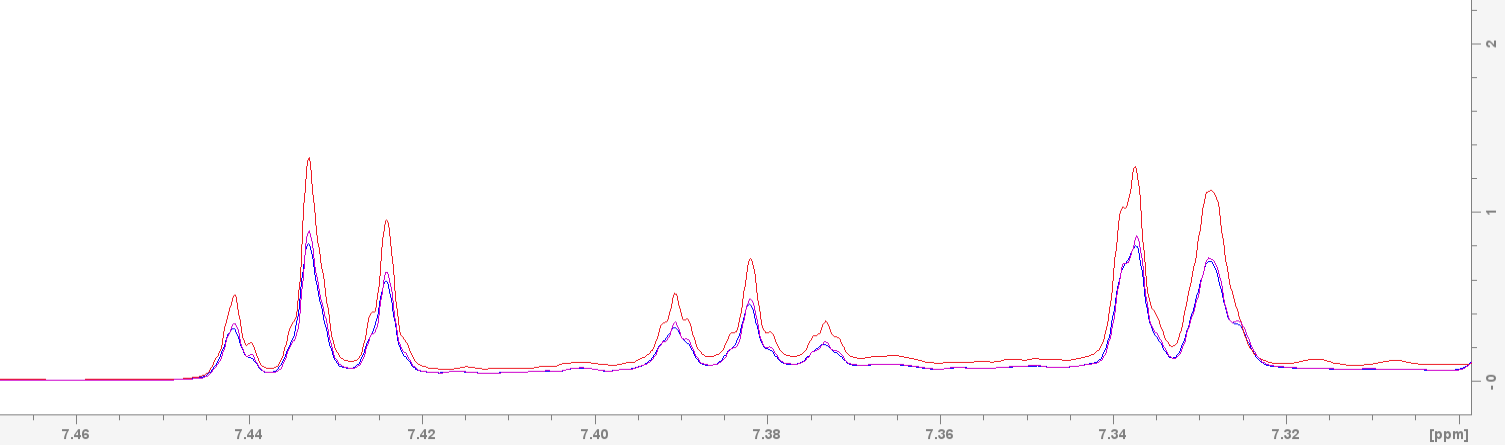


b)


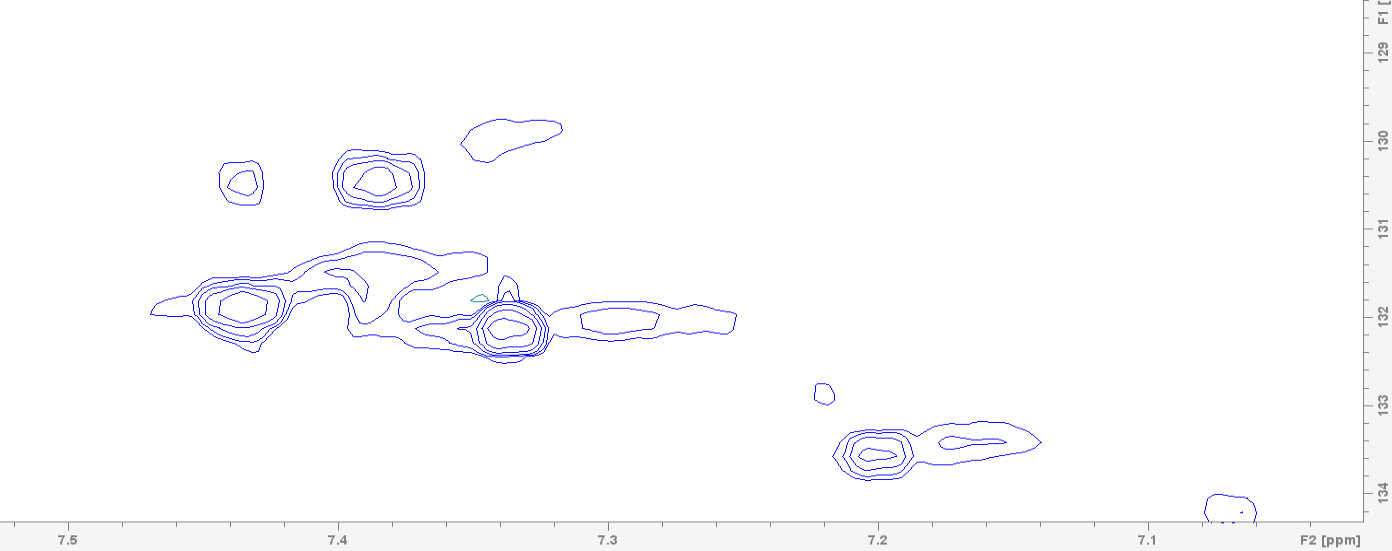


**Fig. S14**: a) 1D spectral overlay of L-phenylalanine at 7.44 - 7.42 ppm, 7.39 - 7.37 ppm and 7.34 - 7.33 ppm produced by spores germinated with TSB germinant solution. b) 2D signal of L-phenylalanine at 7.44 - 7.42 ppm, 7.39 - 7.37 ppm and 7.34 - 7.33 ppm on the proton frequency and 131, 130 and 132 ppm on the carbon frequency produced by spores germinated in TSB germinant solution.

*Signals colored (pink, red and blue) represent samples collected at; 3-hour time point, media control and 0-hour time point (before germination), respectively.

a)


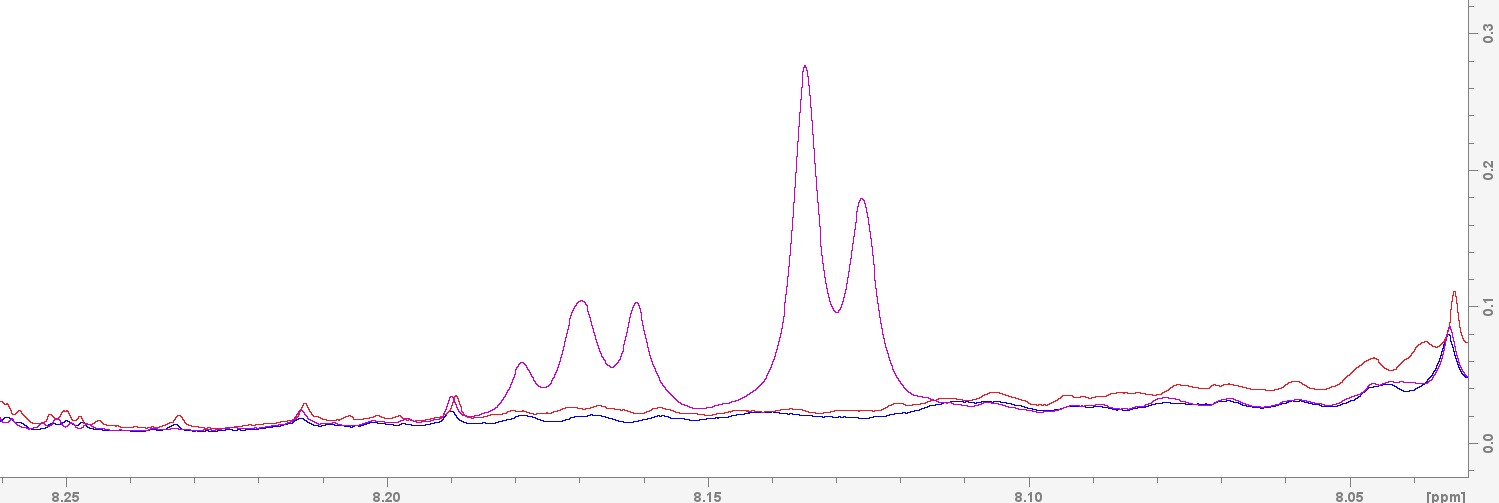


b)


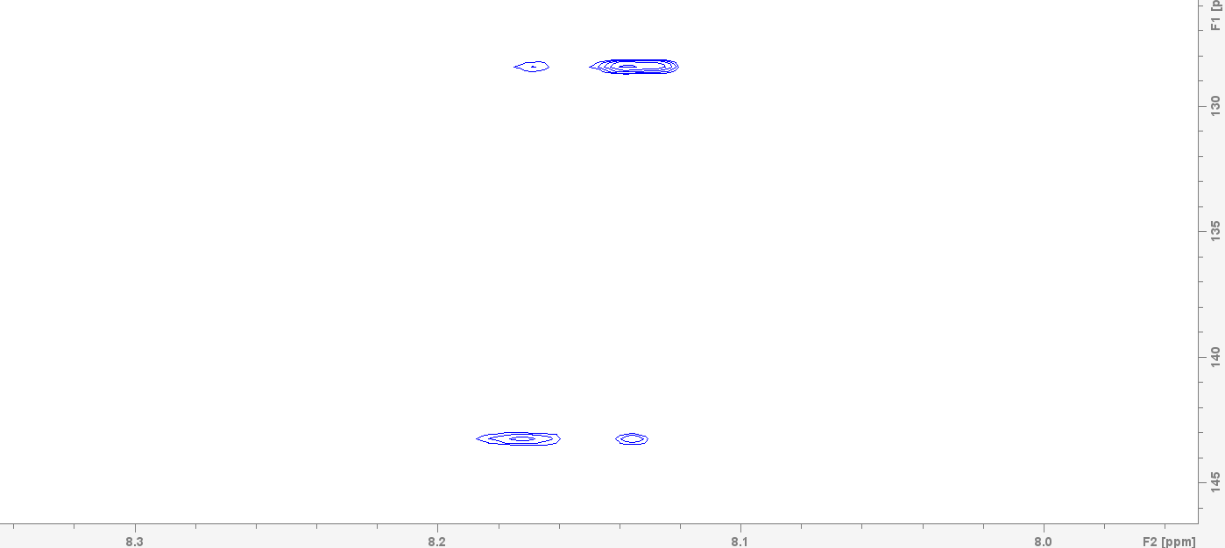


**Fig. S15**: a) 1D spectral overlay of dipicolinic acid at 8.18 - 8.12 ppm produced by spores germinated in TSB germinant solution. b) 2D signal of dipicolinic acid at 8.18 - 8.12 ppm on the proton frequency and 129 and 143 ppm on the carbon frequency produced by spores germinated in TSB germinant solution.

*Signals colored (pink, red and blue) represent samples collected at; 3-hour time point, media control and 0-hour time point (before germination), respectively.


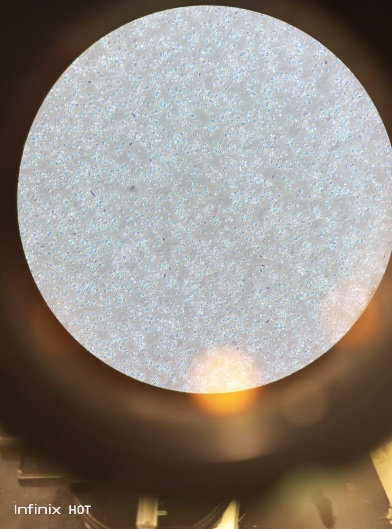

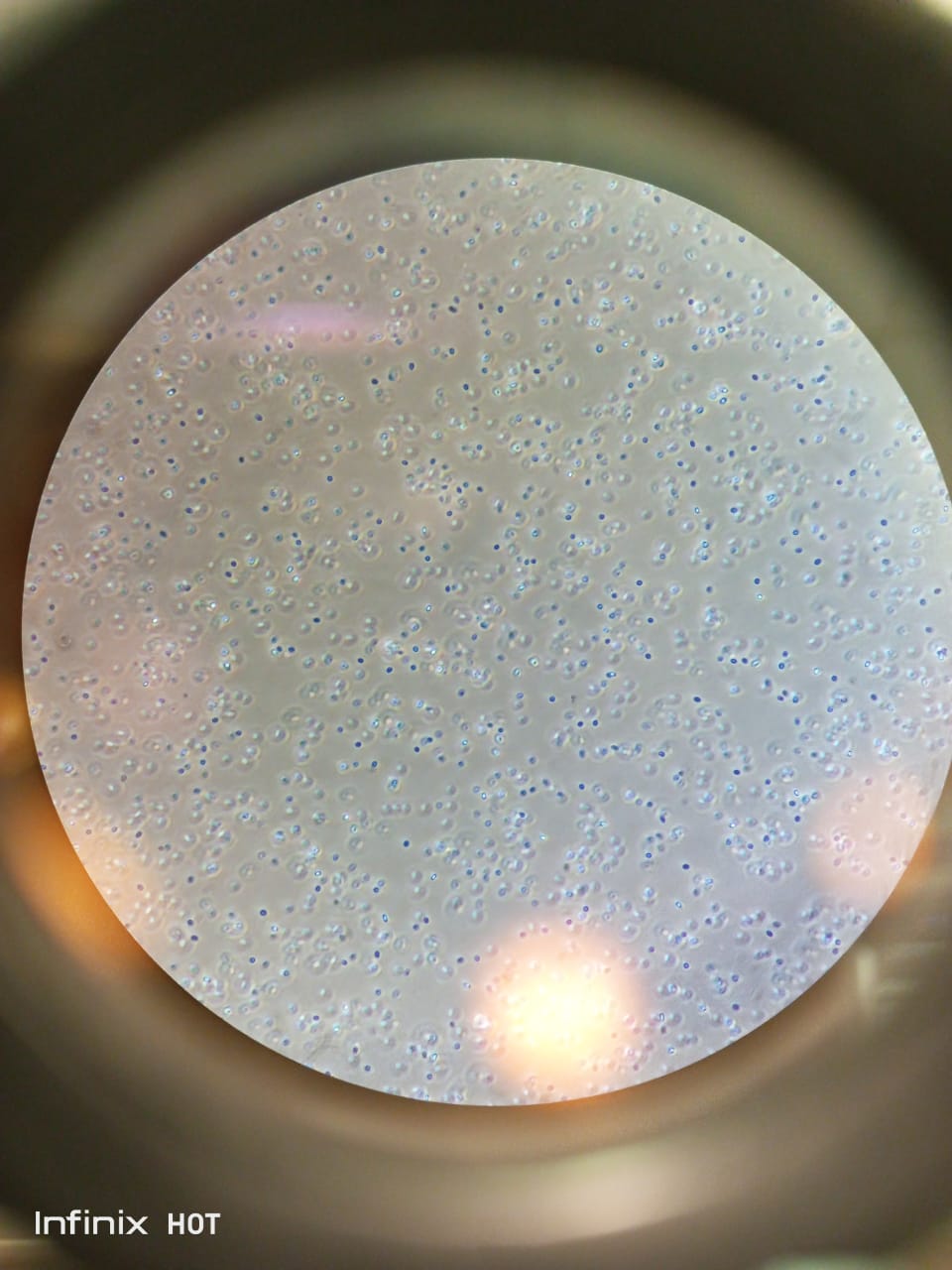

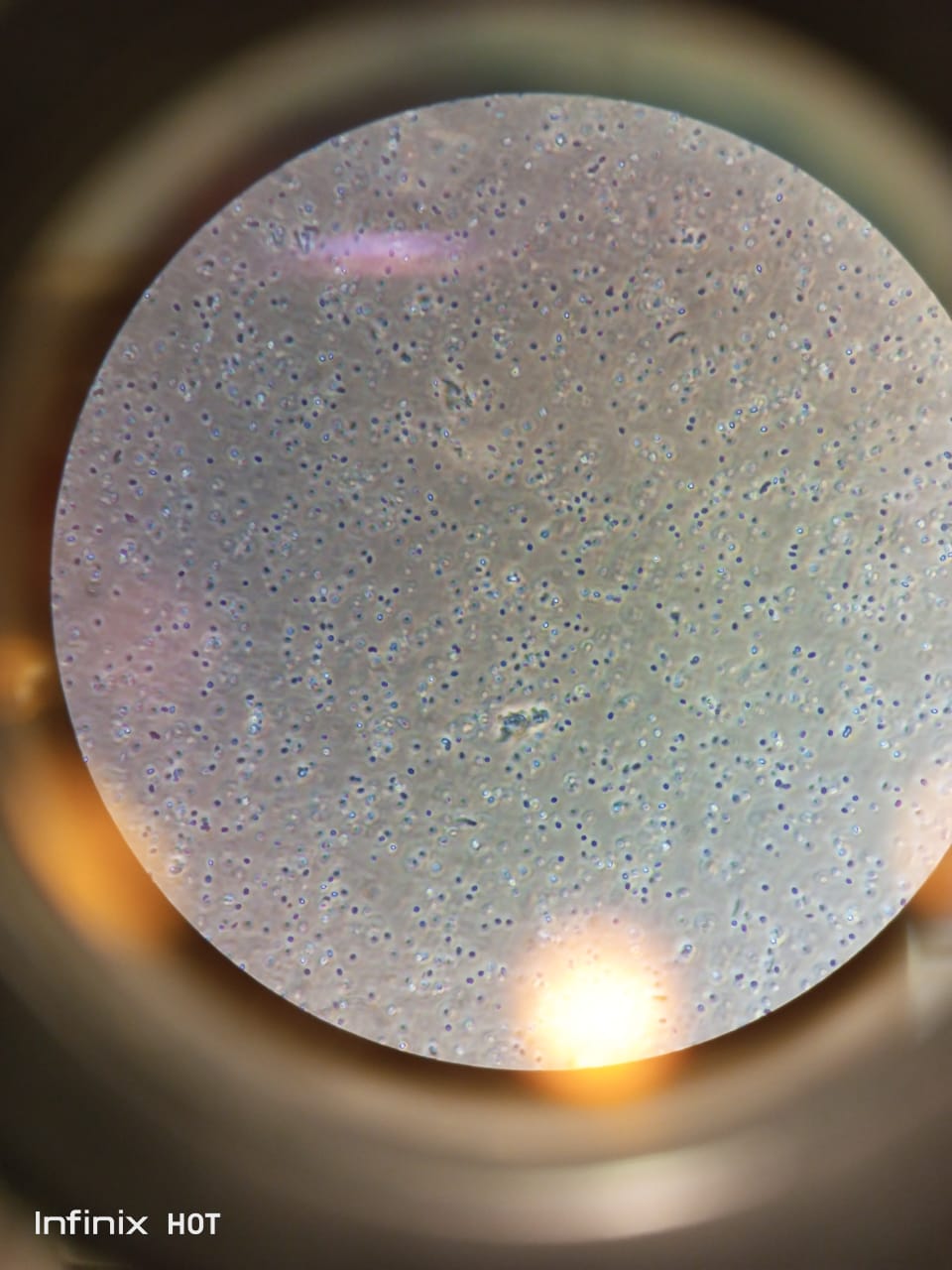

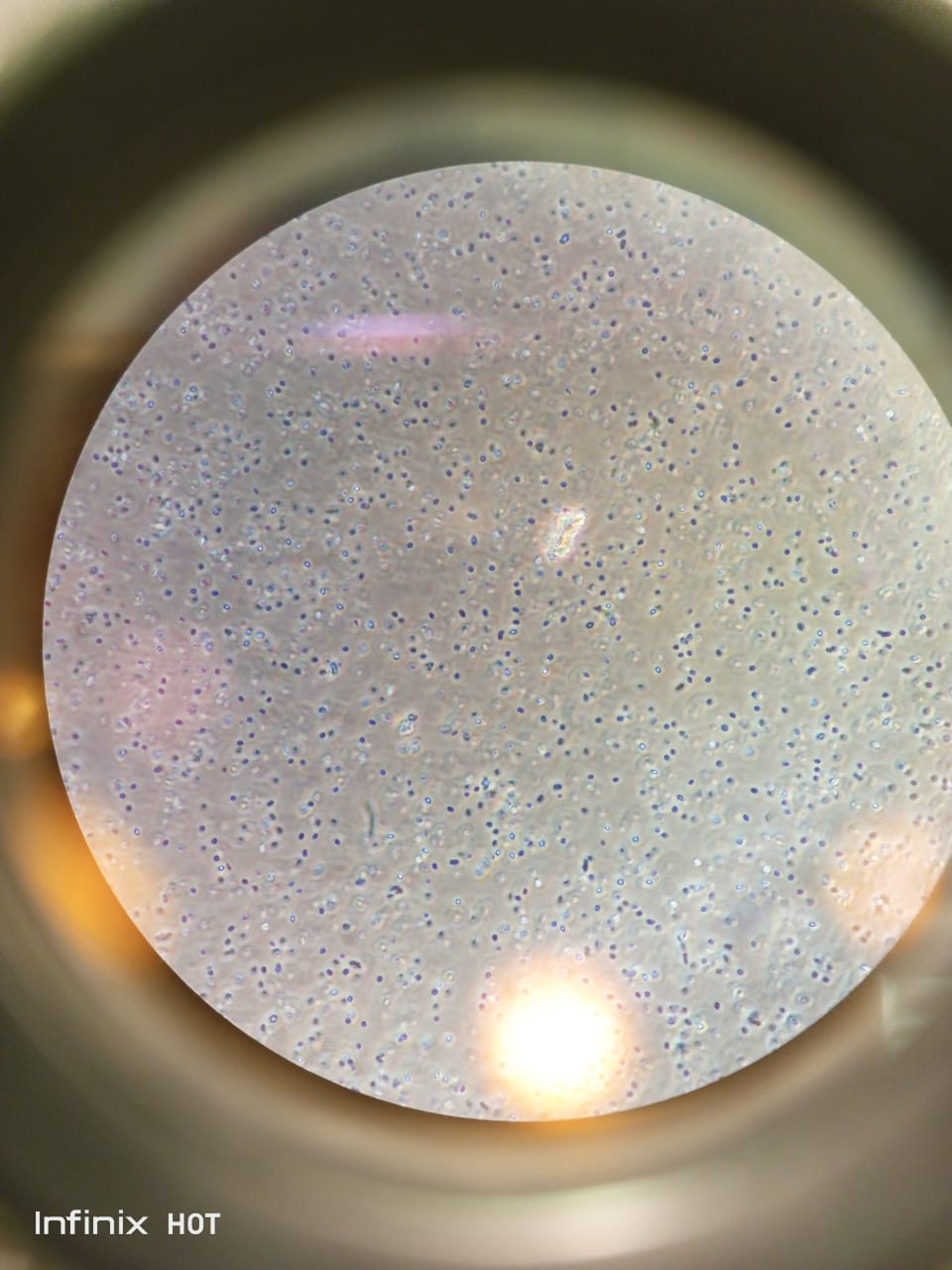

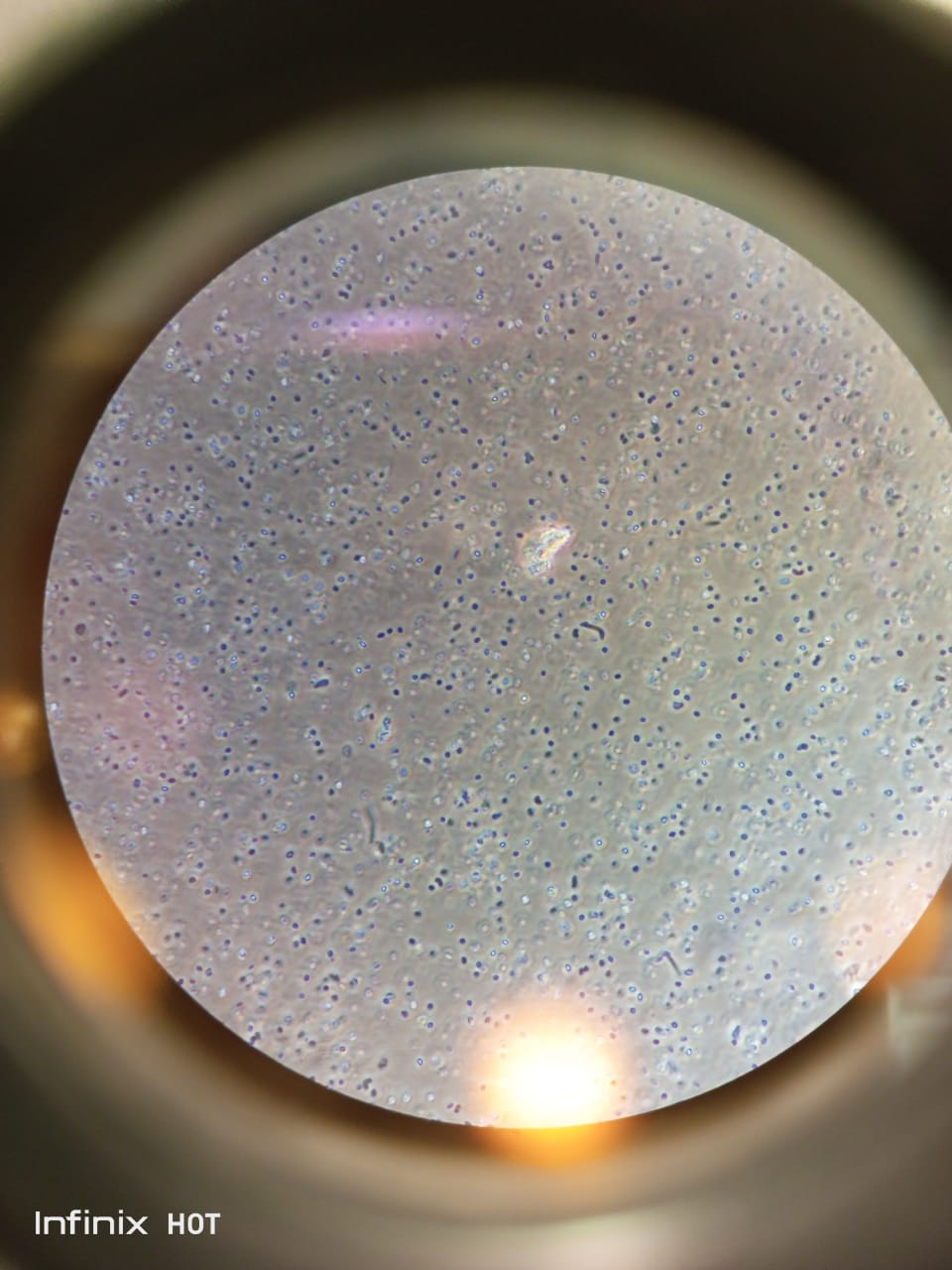


**0 hour**

**1 hour**

**2 hours**

**3 hours**

**4 hours**

**A. TSB-induced Germination**

**B. AGFK-induced Germination**


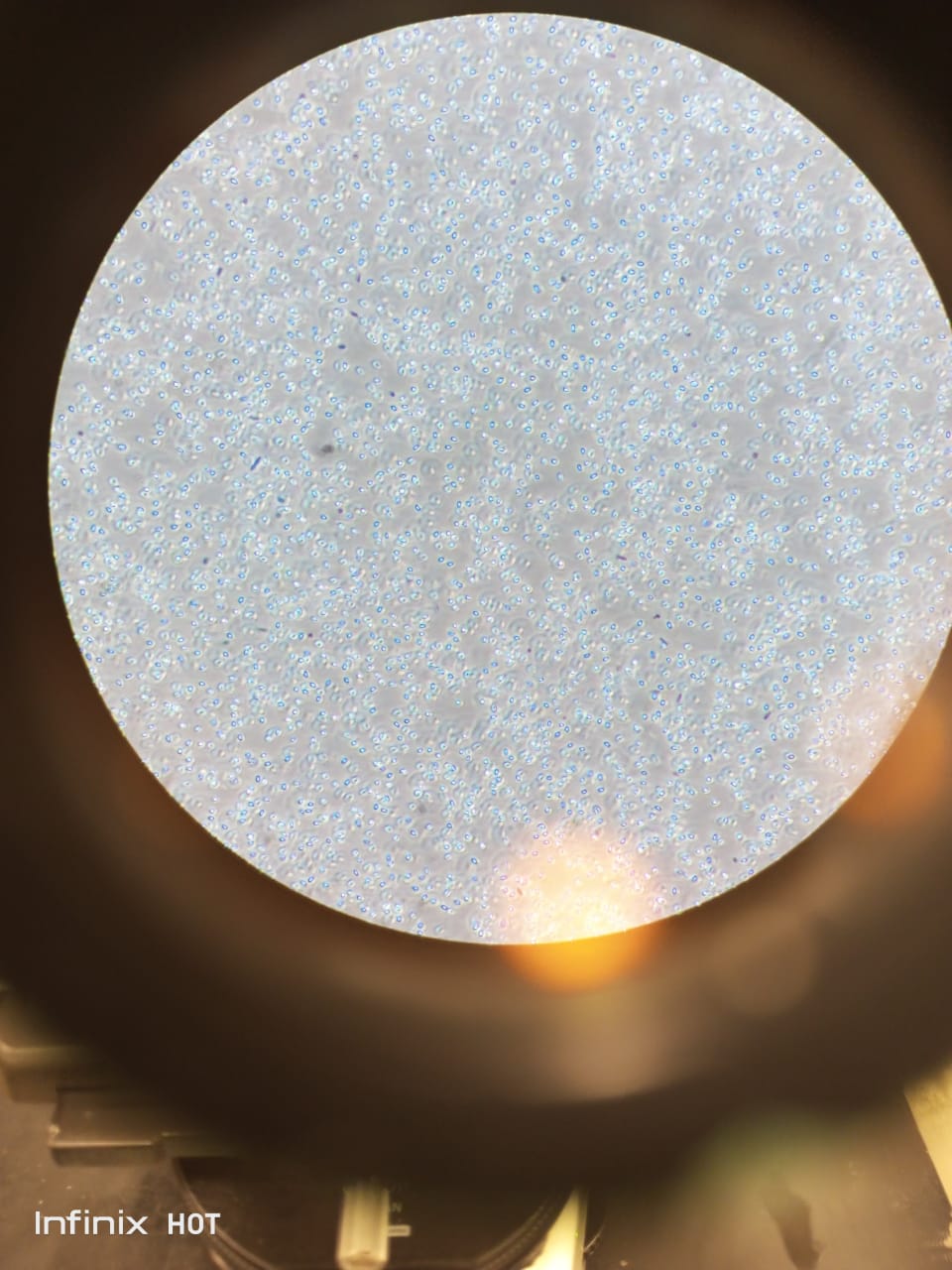

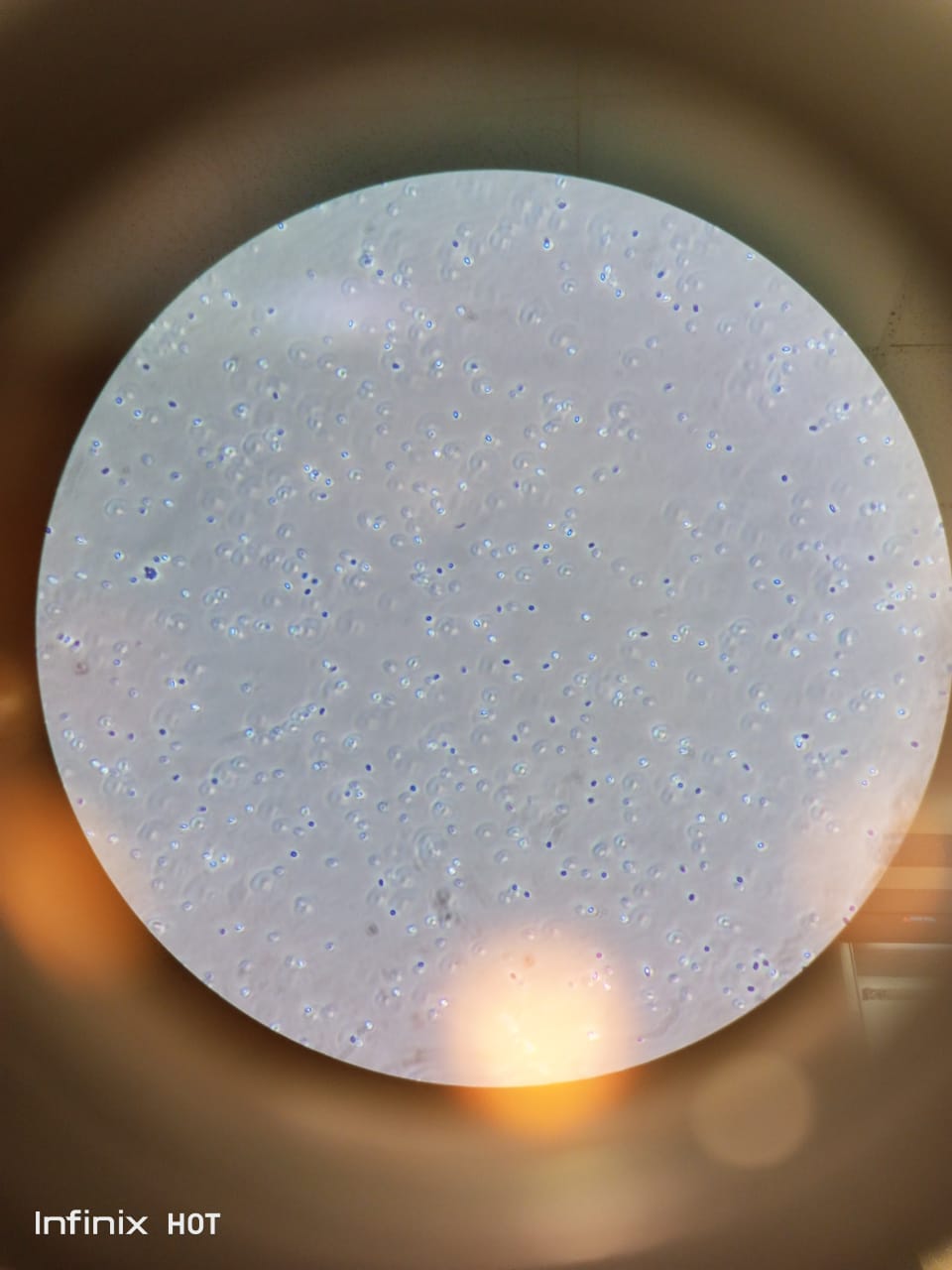

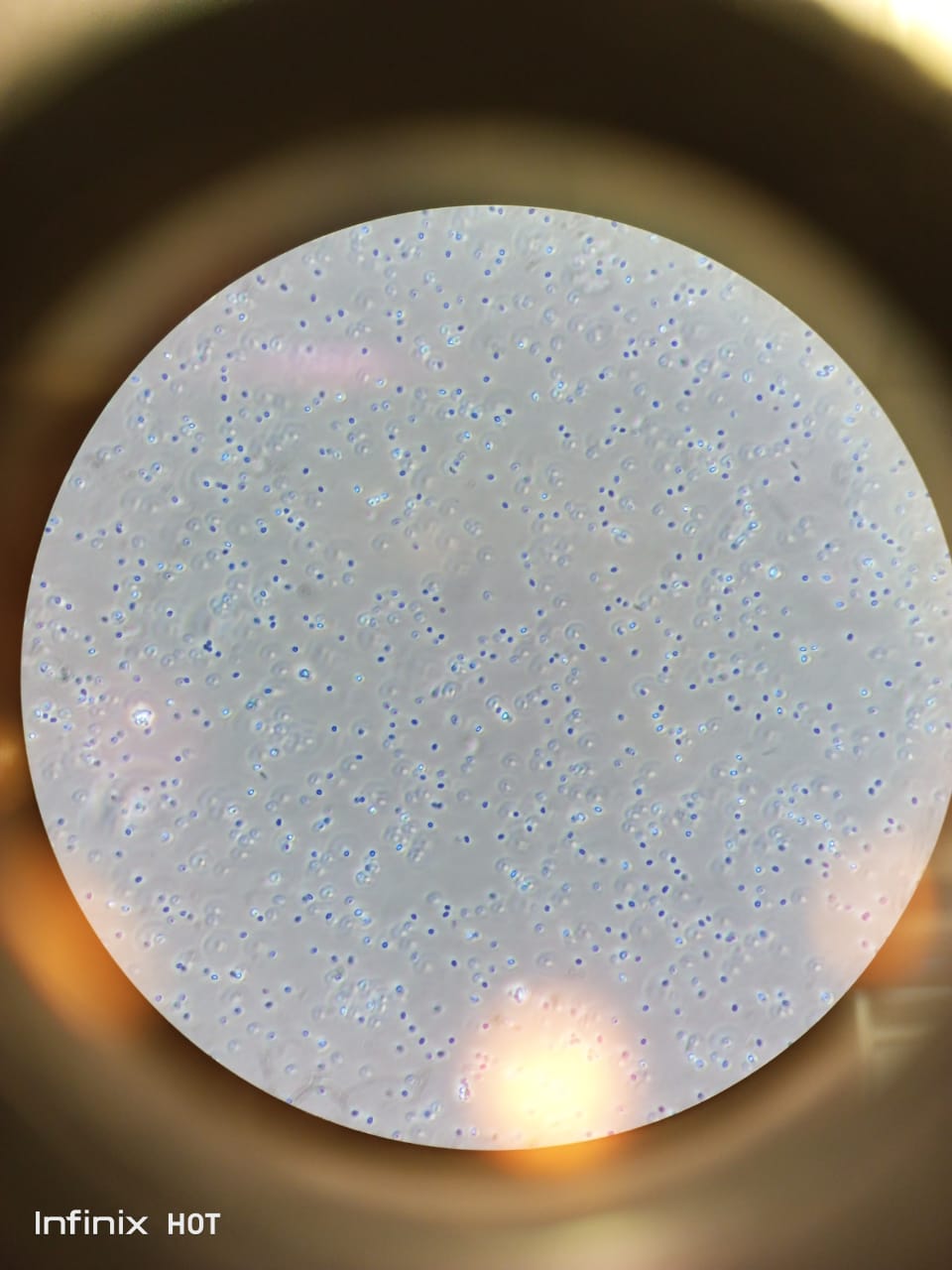


**0 hour**

**1 hour**

**2 hours**


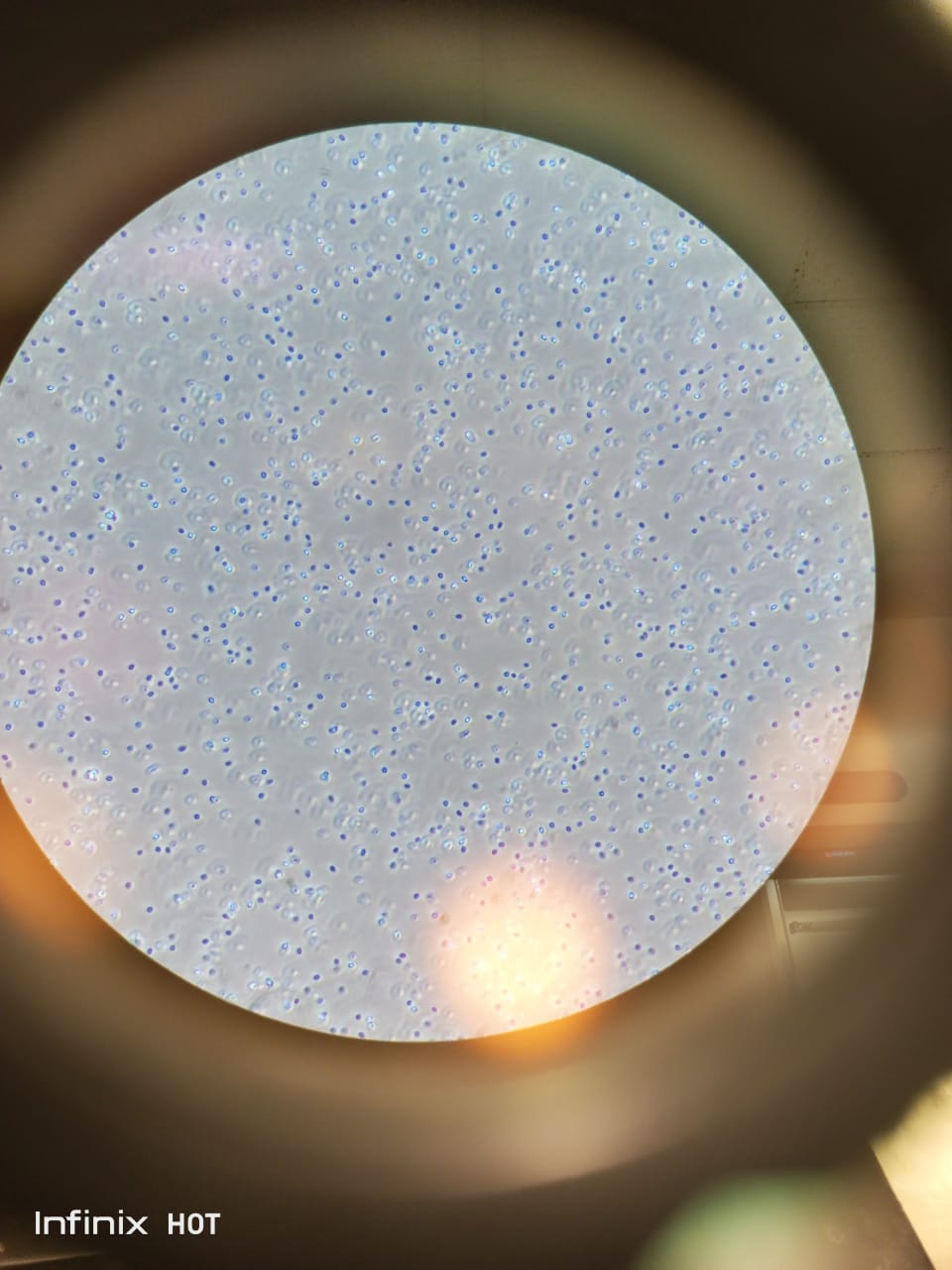


**3 hours**


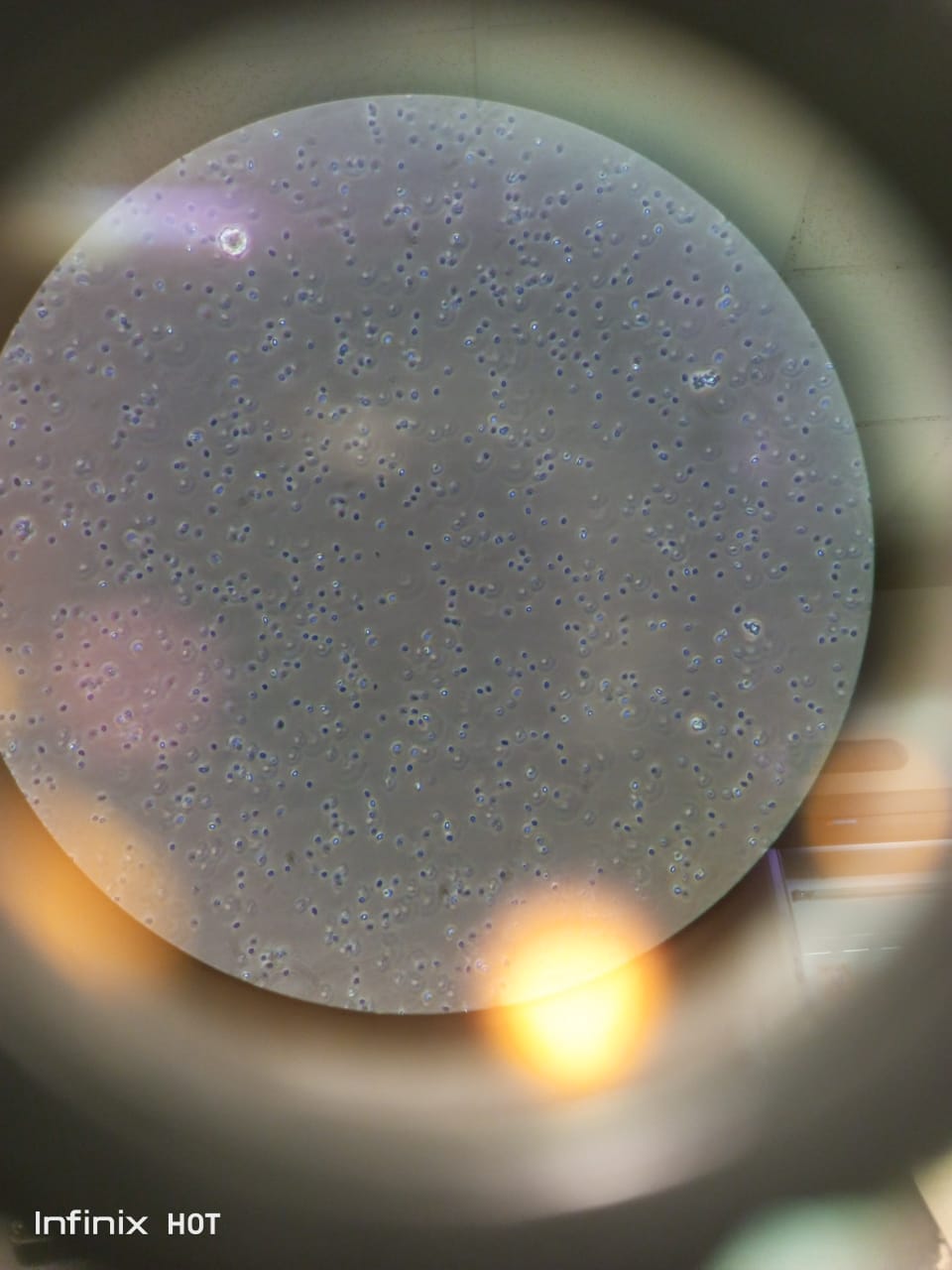


**4 hours**

**Fig. S16:** Phase Contrast Microscopic Pictures for 0-hour through 4-hour time periods. A: TSB, B: AGFK.
